# Formation and Properties of a Bicyclic Silylated Digermene

**DOI:** 10.1002/chem.201402785

**Published:** 2014-06-30

**Authors:** Johann Hlina, Judith Baumgartner, Christoph Marschner, Lena Albers, Thomas Müller, Viatcheslav V Jouikov

**Affiliations:** [a] Institut für Anorganische Chemie, Technische Universität GrazStremayrgasse 9, 8010 Graz (Austria) E-mail: christoph.marschner@tugraz.at; [b] Institut für Chemie, Universität GrazStremayrgasse 9, 8010 Graz (Austria) E-mail: baumgartner@tugraz.at; [c] Institut für Chemie, Carl von Ossietzky Universität Oldenburg26111 Oldenburg (Federal Republic of Germany); [d] UMR 6226, Chimie et Photonique Moléculaires, Université de Rennes 135042 Rennes (France)

**Keywords:** digermenes, electrochemistry, germylene, radicals, silicon

## Abstract

In the presence of PMe_3_ or N-heterocyclic carbenes, the reaction of oligosilanylene dianions with GeCl_2_⋅dioxane gives germylene–base adducts. After base abstraction, the free germylenes can dimerize by formation of a digermene. An electrochemical and theoretical study of a bicyclic tetrasilylated digermene revealed formation of a comparably stable radical anion and a more reactive radical cation, which were characterized further by UV/Vis and ESR spectroscopy.

## Introduction

In recent years, the chemistry of the heavier carbene[1–5] and alkene[6–14] analogues has become an intensely studied field. Although much of the research in this particular area concentrates on the elements silicon and tin, germanium is also becoming increasingly popular. A simple way of classifying these compounds is according to substituent types. Starting with Lappert’s seminal work, π-basic (donating) substituents such as N(SiMe_3_)_2_[18] or more recently SR,[19] have been recognized as suitable ligands for tetrylenes, which are stabilized by way of π donation into the empty p orbital. One consequence of this stability is a diminished tendency to dimerize to heavy alkene analogues.

Alkyl, aryl, or silyl substituted tetrylenes on the other hand are much more reactive and exhibit a pronounced tendency for dimerization or even oligomerization. The fact that linear or cyclic chains of heavy Group 14 atoms are formally composed of tetrylenes can be utilized to access these species for instance by photochemical methods. In cases with sterically demanding substituents, tetrylenes typically dimerize to heavy alkene analogues.

Reactions of organometallic reagents with divalent heavy Group 14 halides can be considered as an interesting synthetic alternative to tetrylenes and, after dimerization, to heavy alkene formation. A major reason why this strategy is only a rarely employed route may be the incompatibility of free tetrylenes and the strongly nucleophilic organometallic reagents.

Recently, we described the reaction of a 1,4-dipotassiotetrasilane with GeBr_2_⋅dioxane and PEt_3_ to give the phosphane adduct of a germylene embedded into a five-membered cyclosilane ring.[20] The presence of the phosphane as a donor molecule prevents further interaction of the germylene with the potassium silanide. The release of the free germylene can be accomplished by base abstraction with the strong Lewis acid B(C_6_F_5_)_3_. For the case of the five-membered germylene, this resulted in a subsequent 1,2-trimethylsilyl shift to form a silagermene, which eventually dimerized in a [2+2]-cycloaddition reaction.[20]

Herein, we describe the synthesis of related germylene–base adducts which upon being released, dimerize and rearrange to oligocyclic silylated digermenes. The reasons for the fundamentally different reactivity of the two cyclic silylgermylenes can be explained on the basis of theoretical calculations. One digermene was investigated with respect to its redox properties by spectroelectrochemical and theoretical methods.

## Results and Discussion

### Formation

**Scheme 1:**
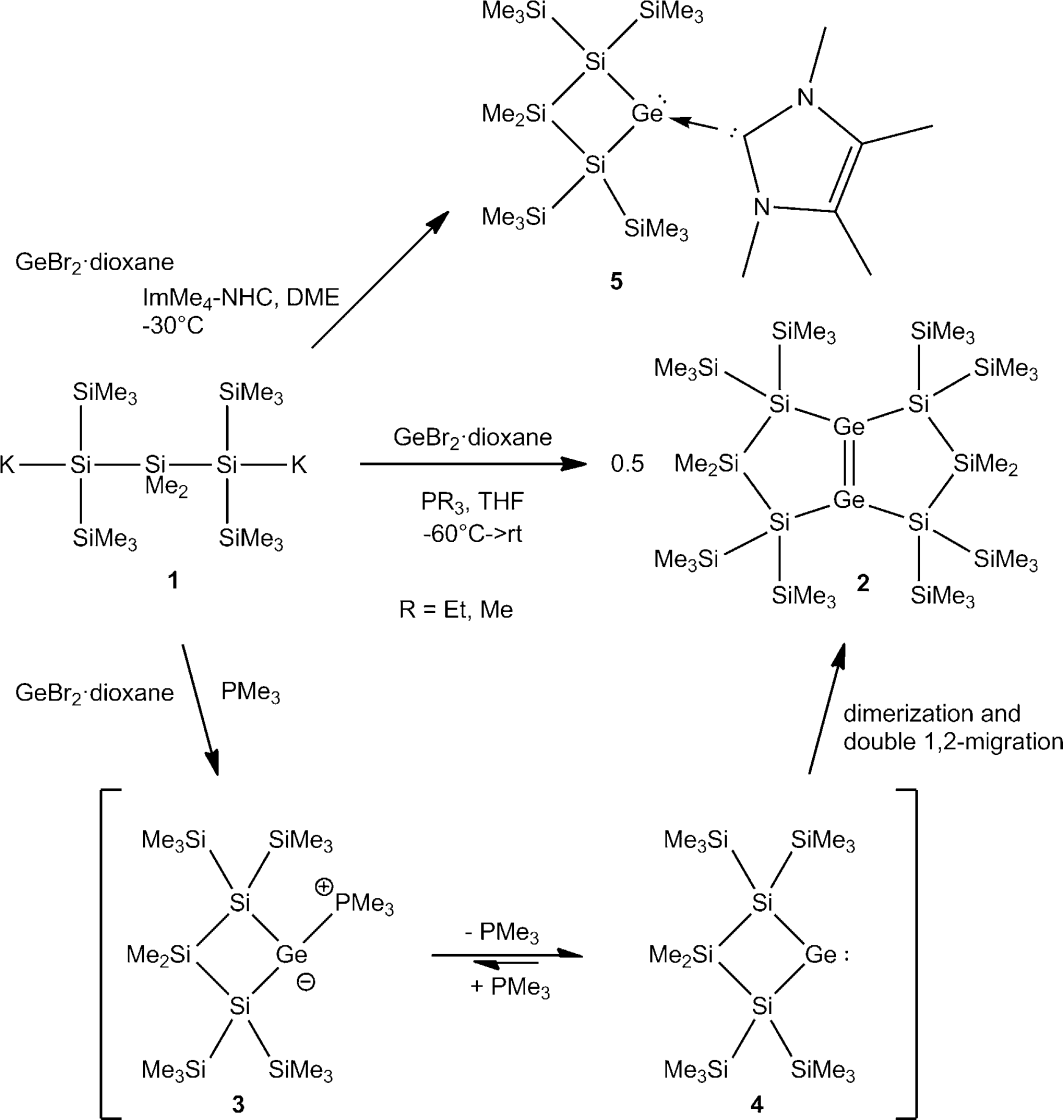
Reactions of 1,3-trisilanylene dianion 1 with GeBr_2_⋅dioxane in the presence of bases.

The reaction of the 1,3-dipotassiotrisilane **1**[21] with GeBr_2_⋅dioxane and PEt_3_ was found to give not the respective germylene base adduct but rather a bicyclic digermene **2** (Scheme 1), in close analogy to what had been observed before by Kira and co-workers for a related disilylated silylene.[22, 23] Change of PEt_3_ for PMe_3_ in the reaction again led to digermene **2**, but in this case, the PMe_3_ adduct **3** of the transient germylene **4** was detected in solution by NMR spectroscopy. Upon removing the solvent in vacuum, **2** formed again. The use of tetramethylimidazol-2-ylidene (IMe_4_) as a base allowed the selective formation of NHC-stabilized germylene **5** (Scheme 1).[24]

**Scheme 2:**
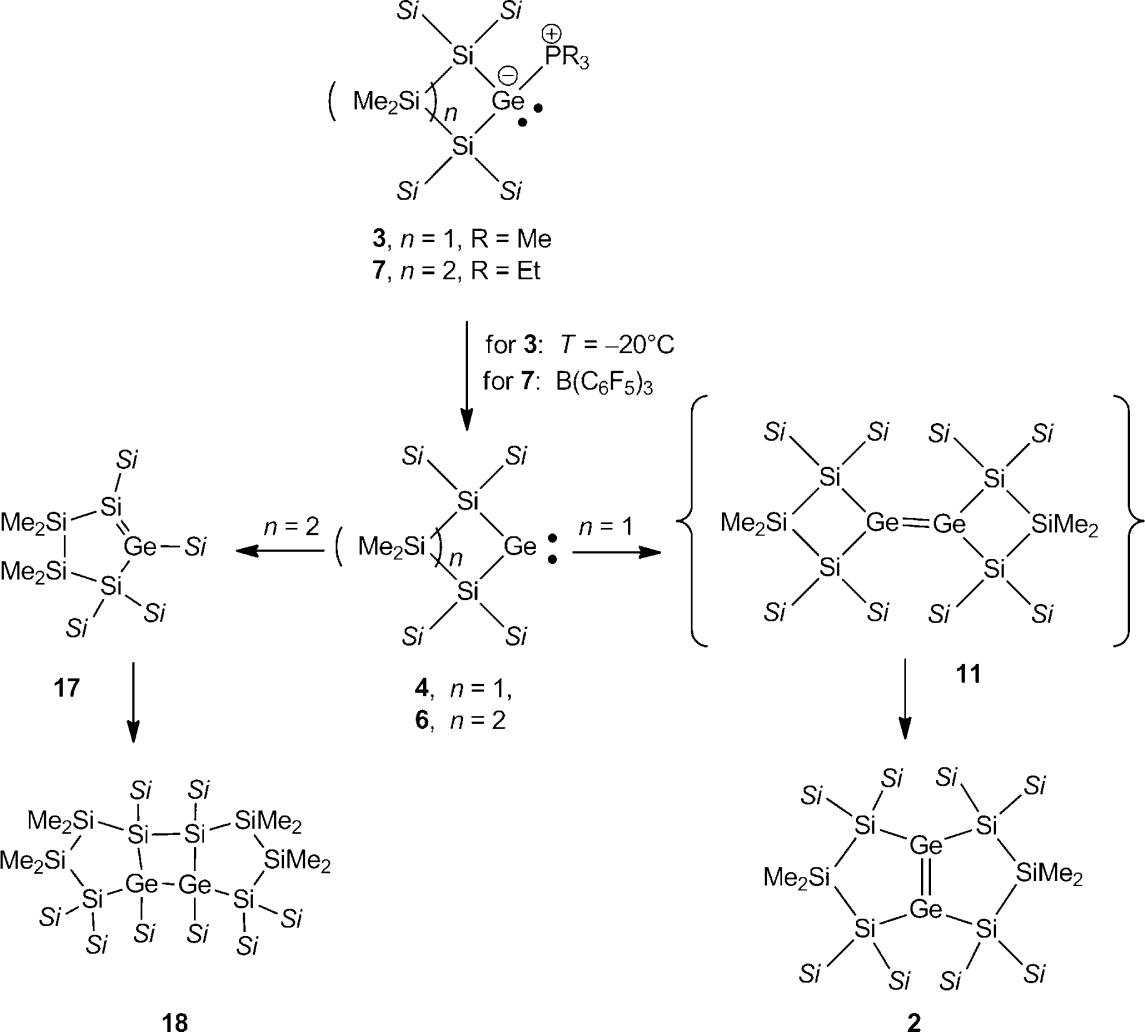
Comparison of the different reactivities of germylene–phosphane complexes 3 and 7[20] (*Si*=SiMe_3_).

The difference between the dimerization reactivity of the four-membered cyclic germylene **4** and the previously disclosed reactivity of its direct homologue, the five-membered cyclic germylene **6**,[20] is striking. Common to both germylenes **4** and **6** is their tendency to form zwitterionic complexes with phosphanes. The complex between germylene **6** and PEt_3_, **7**, is stable at room temperature and an isolable compound (Scheme 2).[20] Without stabilization by an additional Lewis base, however, germylene **6** underwent a 1,2-silyl shift to give cyclic silagermene **17**, which dimerizes to give the tricyclic polysiladigermane **18** (Scheme 2).[20]

In contrast, the PMe_3_ complex of the four-membered cyclic germylene (**3**), is not stable at temperatures as low as −20 °C. At this temperature, germylene **4** dimerizes to give, after skeletal rearrangement, bicyclic digermene **2** (Scheme 1 and 2). We used density functional calculations[25] to provide some understanding of this surprising difference between the reactivity of four-membered cyclic germylene **4** and that of its five-membered homologue, **6**.

**Figure 1:**
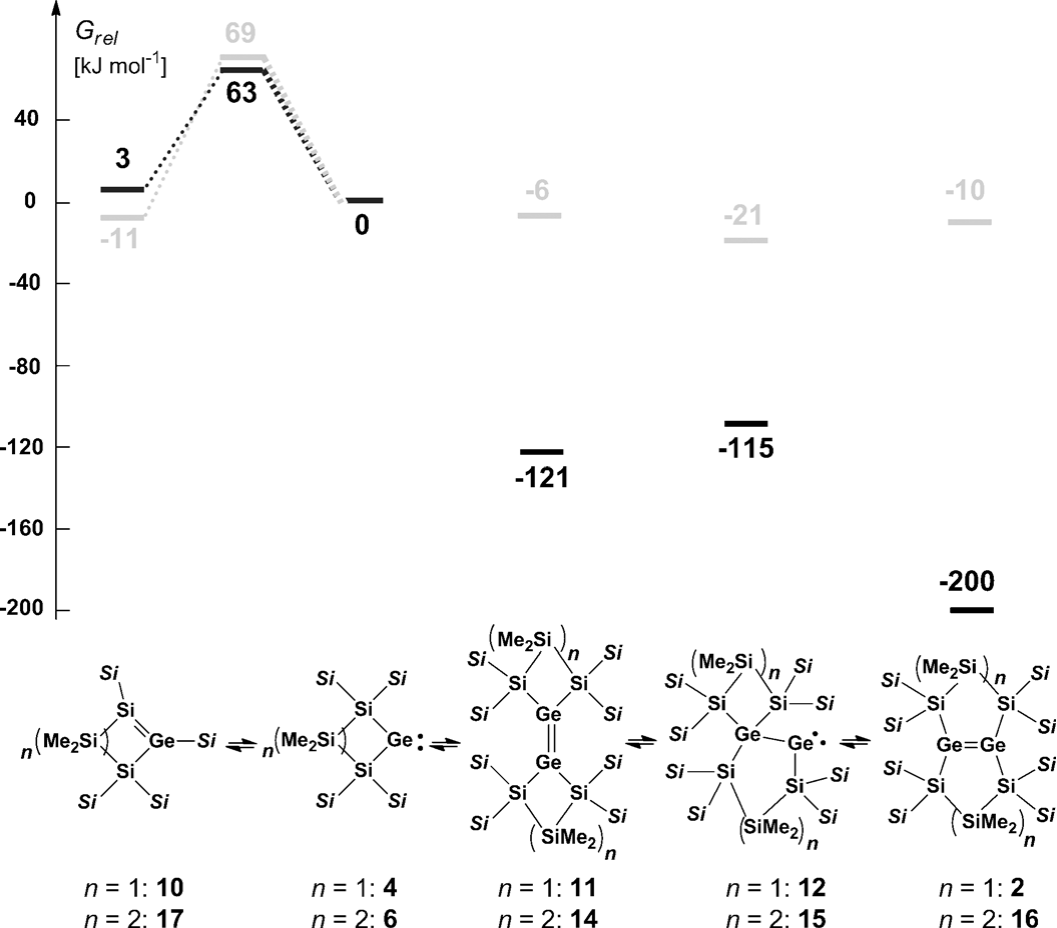
Relative free Gibbs enthalpies at 253 K for germylene 4, silagermene 10, and digermene 2 and its isomers 11, 12, as calculated at M062X/6-311+G(d,p)(Si, C, H) def2-tzvp (Ge) (in black, *Si*=SiMe_3_). For comparison, literature data[20] for the homologous compounds 6 and 14–17 are also shown (at 298 K, in gray).

A possible rearrangement of germylene **4** to the cyclic silagermene **10** is only slightly endergonic and is connected with a free-energy barrier at *T*=253 K of 63 kJ mol^−1^. In this respect, a comparison with homologous germylene **6** reveals no fundamental difference (see Figure 1). The calculated bond dissociation energy (BDE) of the Ge–P bond of PMe3 adduct **3** is however reduced compared to that of related phosphane adduct **7**, in agreement with the observed lower temperature stability of **3**, BDE(Ge–P)=113 kJ mol^−1^ (**3**) versus 130 kJ mol^−1^ (**7**).[20] The increased thermal instability of adduct **3** delivers germylene **4** at temperatures as low as −20 °C. At these temperatures, the silylgermylene/silagermene rearrangement to give silagermene **10** (Figure 1) is relatively slow.[26] This favors the competing practically barrier free dimerization of germylene **4** to give digermene **11** (Scheme 2, Figure 1).

**Figure 2:**
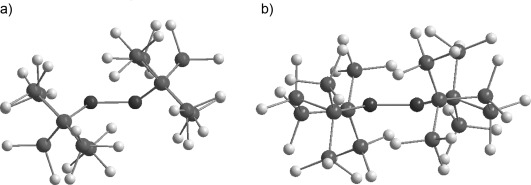
Ball and stick representation of the calculated molecular structures of digermenes. a) 11; selected calculated structural parameter: Ge–Ge=231.5 pm, Si-Ge-Si 90.8°; *trans–*bent angle β: 35.6°; b) 14; selected calculated structural parameter: Ge–Ge=233.8 pm, Si-Ge-Si 105.3°; *trans–*bent angle β: 11.3° (color code: black (Ge), dark gray (Si), light gray (C), hydrogen atoms are not shown. Calculated at M062X/6-311+G(d,p) (Si,C,H);def2tzvp(Ge)).

After double silyl migration via germylgermylene **12**, the thermodynamically most stable compound in this series, digermene **2**, is obtained (see Figure 1). The results of the calculations indicate that dimerization of germylene **4** to digermene **11** has a strong thermodynamic driving force as it is favored by Δ*G*^253^=−121 kJ mol^−1^. In contrast, the corresponding dimerization of germylene **6** is practically thermoneutral (Δ*G*^298^=−6 kJ mol^−1^, see Figure 1).[20] One reason for this difference might be the electronic structure of the germylenes and, as a consequence, the structures and stabilities of the respective dimers. Substitution with electropositive silyl groups leads to a smaller energy separation between the singlet and triplet states of the germylene, Δ*E*_ST_.[10] In agreement with established theoretical models,[27–29] almost planar tetrasilyl digermenes with relative short Ge=Ge bonds (Ge–Ge=226.7–229.8 pm) are formed upon their dimerization.[30] The acute Si-Ge-Si bond angle of four-membered cyclic germylene **4** increases Δ*E*_ST_, for this germylene compared to that of five-membered cyclic germylene **6** or to related acyclic germylene ((Me_3_Si)_3_Si)_2_Ge: **13** (**4**: *α*(Si-Ge-Si): 80.6°, Δ*E*_ST_=102 kJ mol^−1^; **6**: *α*(Si-Ge-Si): 93.6°, Δ*E*_ST_=86 kJ mol^−1^; **13**: *α*(Si-Ge-Si)=112.1, Δ*E*_ST_=71 kJ mol^−1^). The higher Δ*E*_ST_ for germylene **4** results in a molecular structure of Ge=Ge bonded dimer **11** that has a significant *trans*–bent arrangement (Figure 2).[31] This *trans*–bent conformation efficiently separates the bulky silyl substituents at the two different germanium atoms of **11** and stabilizes germylene dimer **11** relative to its constituent monomers. In contrast, the smaller Δ*E*_ST_ of germylene **6** induces only a small *trans*-bending of digermene **14** (Figure 2) and, consequently, the Ge=Ge bond is weakened by the steric repulsion of the neighboring silyl groups. A structural indication for this scenario is provided by the fact that the *trans*–bent distorted Ge=Ge bond of digermene **11** is shorter than the almost-planar Ge=Ge bond of digermene **14**, a reversal of the expected and previously documented trend.[32]

**Scheme 3:**
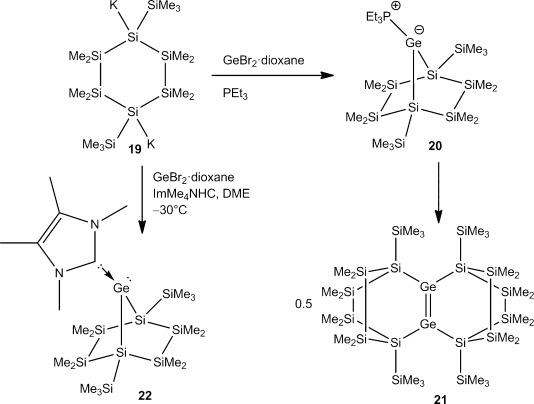
Reactions of cyclic 1,4-cyclohexasilanylene dianion 19 with GeBr_2_⋅dioxane in the presence of bases.

The reaction of 1,4-dipotassiocyclohexasilane **19**[33] with GeBr_2_⋅dioxane and PEt_3_ proceeded in a similar way, but germylene–phosphane adduct **20** (Scheme 3) was found to be slightly more stable than **3** and was characterized by multinuclear NMR spectroscopy. Attempts to crystallize the compound eventually led to the isolation of tetracyclic digermene **21**. The same reaction in the presence of carbene IMe_4_ gave stable NHC–germylene adduct **22** (Scheme 3).

### Crystallography

**Figure 3:**
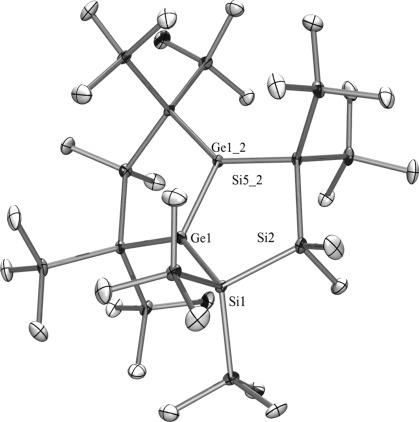
Crystal structure of 2. Thermal ellipsoids are represented at the 30 % level and hydrogen atoms have been omitted for clarity. Ge(1)–Ge(1_2) 2.2665(11), Ge(1)–Si(1) 2.3662(13), Ge(1)–Si(5_2) 2.3714(16), Si(2)–Si(1) 2.359(2), Ge(1_2)-Ge(1)-Si(1) 108.70(5), Ge(1_2)-Ge(1)-Si(5) 111.28(6), Si(1)-Ge(1)-Si(5) 139.98(5), Si(1)-Si(2)-Si(5_2) 112.56(7).

Compounds **2**, **21**, and **22** were subjected to single-crystal structure analysis. As expected, the digermene **2** (Figure 3) is isostructural to the disilene previously reported by Kira and co-workers.[22] The Ge–Ge double bond of 2.2663(9) Å is slightly shorter than other tetrasilylated digermenes reported so far.[30, 34] Also the Si–Ge bonds of 2.371(2) and 2.366(1) Å are unusually short and in the same range as the Si–Si bonds in the molecule. While **2** has a very small *trans*–bent angle of 2.5° the twisting angle between the disilylated germylene units, *τ*, is 16.2°.

**Figure 4:**
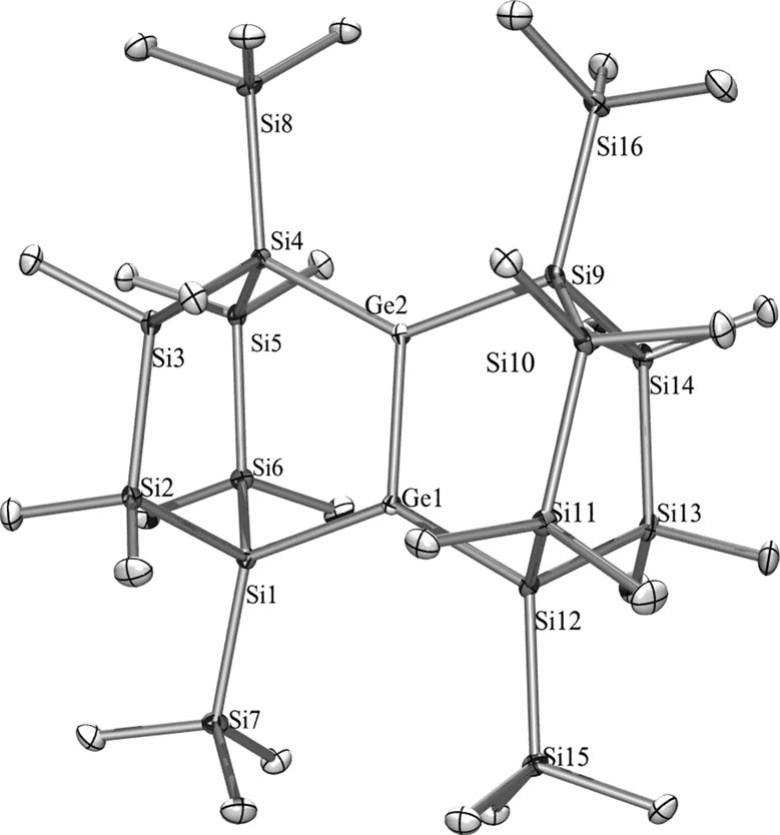
Crystal structure of 21. Thermal ellipsoids are represented at the 30 % level and hydrogen atoms have been omitted for clarity. Ge(1)–Ge(2) 2.2897(6), Ge(1)–Si(12) 2.3836(8), Ge(1)–Si(1) 2.3902(9), Ge(2)–Si(4) 2.3838(8), Ge(2)–Si(9) 2.3913(9), Si(1)–Si(2) 2.3415(9), Ge(2)-Ge(1)-Si(12) 111.563(19), Ge(2)-Ge(1)-Si(1) 113.000(17), Si(12)-Ge(1)-Si(1) 134.93(3), Ge(1)-Ge(2)-Si(4) 111.915(19), Ge(1)-Ge(2)-Si(9) 112.613(17), Si(4)-Ge(2)-Si(9) 135.44(3).

For related digermene **21** (Figure 4), which is sterically more crowded, all bonds are slightly elongated compared to **2**. This is shown by a Ge–Ge double bond length of 2.2896(6) Å and by Si–Ge bonds ranging from 2.3836(6) to 2.3914(8) Å, which are still unusually short. The Si–Si distances cover a rather typical range from 2.339 to 2.356 Å. The diminished twisting angle between the germylene units, *τ*, of 5.2°, and the *trans–*bent angles of 8.3° and 2.1° indicate that the digermene unit of **21** is almost planar. Twisting and *trans–*bent angles of **2** and **21** are in accordance to Kira’s tetrasilylated digermenes.[30]

**Figure 5:**
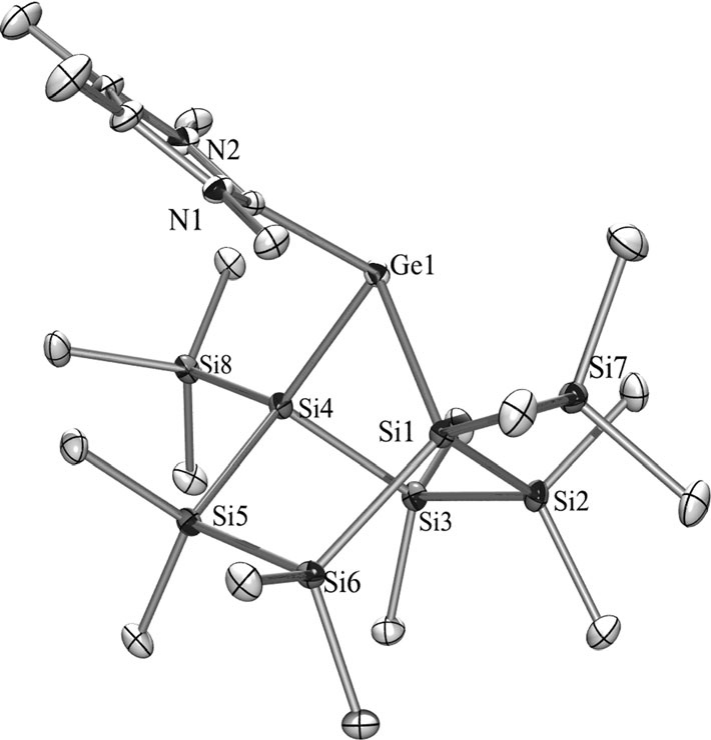
Crystal structure of 22. Thermal ellipsoids are represented at the 30 % level and hydrogen atoms have been omitted for clarity. Ge(1)–C(1) 2.055(2), Ge(1)–Si(4) 2.4817(7), Ge(1)–Si(1) 2.4861(7), N(1)–C(1) 1.343(3), N(2)–C(1) 1.361(3), Si(1)–Si(2) 2.3500(9), C(1)-Ge(1)-Si(4) 96.75(6), C(1)-Ge(1)-Si(1) 107.51(6), Si(4)-Ge(1)-Si(1) 86.88(2), C(21)-Si(8)-Si(4) 108.75(8), N(1)-C(1)-N(2) 104.21(17).

Bicyclic NHC–germylene adduct **22** (Figure 5) very much resembles the previously published NHC adduct of the monocyclic five-membered disilylated germylene.[20] The Ge–C distance of 2.055(2) Å and the Ge–Si distances of 2.4817(7) and 2.4861(7) Å are almost identical to the respective values of the previously published adduct, 2.071(3), 2.4709(9), and 2.4795(9) Å.[20] The same is true for the angles between the Si-Ge-Si plane and the Ge–NHC bond, which is close to 108° for **22** and about 105° for the five-membered ring.

### NMR spectroscopy

The NMR spectroscopic comparison of compounds **2**, **3**, and **5** with **20**, **21**, and **22** is interesting as these correspond to two sets of disilylated germylene adducts with different oligosilanylene backbones. The most informative nucleus to discuss with respect to this is of course ^29^Si (Table 1). The resonances for **2** are observed at −9.7 (SiMe_3_), −10.5 (SiMe_2_), and −92.7 (Si^q^) ppm, with the SiMe_3_ and SiMe_2_ resonances being almost identical to Kira’s analogous silylene (−8.7, −11.4, −107.1 ppm).[30] Digermene **21** shares with **2** the downfield-shifted resonances for the quaternary silicon atoms, which are attached to the double-bonded germanium atoms.

**Table 1:** Ambient temperature ^29^Si NMR chemical shifts

Compound	Si^q^	SiMe_2_	SiMe_3_
**2**	−92.7	−10.5	−9.7
**3**	−106.3	−10.1	−7.7
**5**	−103.5/−102.1^[a]^	−17.8/−17.3^[a]^	n.o./−9.3^[a]^
**20**	−105.2	−28.7/−33.1	−6.7
**21**	−83.9	−41.0	−6.2
**22**	−105.7	−29.6/−34.3	−6.0

[a] Measured at 60 °C. n.o=not observed.

Compounds **3**, **5**, **20**, and **22** are all germylene adducts with either PMe_3_ (**3**, **20**) or IMe_4_ (**5**, **22**) as the base. The ^29^Si resonances of **20** and **22**, which share the bicyclo[2.2.1] backbone, are very similar (Table 1). Chemical shifts around −6 ppm are typical for bridgehead-connected SiMe_3_ groups. The fact that for **20** and **22** two resonances were observed for the SiMe_2_ groups indicates configurational stability of the germylene atoms. On the other hand, there is a difference between compounds **3** and **5** in that respect. For **3** at ambient temperature, only one resonance for the SiMe_3_ groups was observed, whereas for **5**, at the same temperature, no SiMe_3_ resonance was observed for reasons of coalescence and only at 60 °C a sharp signal was detected. This result clearly indicates a lack of configurational stability of the germanium atom at room temperature for both compounds, but also a higher inversion barrier for NHC-stabilized **5**.

### Spectroelectrochemistry

The possibility of one-electron reduction and oxidation of silylated tetrylenes and their dimerization products has been demonstrated a few times. In particular, Sekiguchi and co-workers have shown elegant examples for disilenes[35, 36] and distannenes.[37]

Real-time ESR- and UV-coupled spectroelectrochemistry can provide important information on mechanism of formation of multiple reduced and oxidized states upon electron transfer(s), on their structure, electron-density distribution, and reactivity. We therefore undertook such studies, which are thus far unprecedented for this type of compounds.[38]

**Figure 6:**
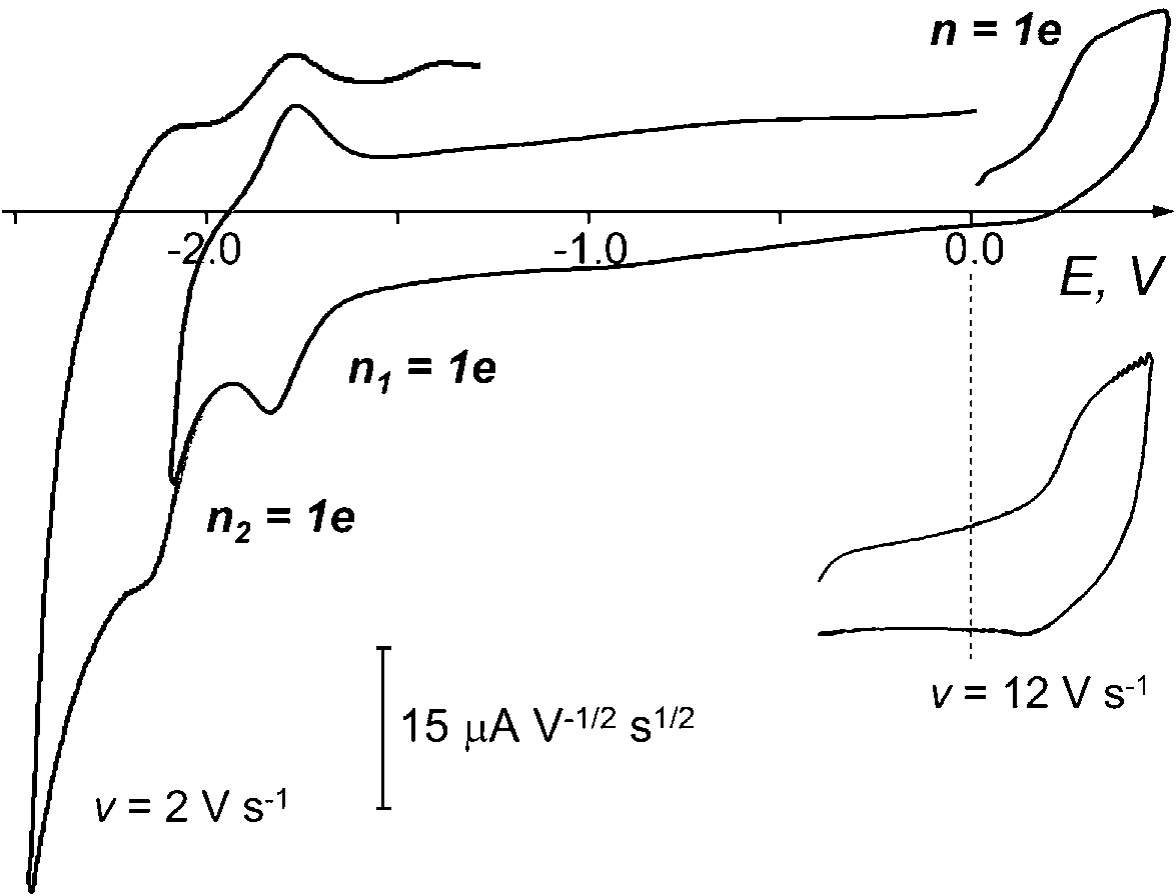
Voltammetry of digermene 2 (3.5 mmol l^−1^) at a GC microdisk electrode in DME/0.1 m Bu_4_N[B(C_6_F_5_)_4_]. *T*=295 K.

The UV/Vis absorption bands of neutral digermene **2** at *λ*=330 nm (7.6×10^3^ M^−1^ cm^−1^) and 470 nm (1.3×10^4^ M^−1^ cm^−1^) are almost identical to the absorptions of Kira’s isostructural disilene[30] at *λ*=328 nm (8.2×10^3^ M^−1^ cm^−1^) and 469 nm (1.4×10^4^ M^−1^ cm^−1^). Compared to disilenes, digermenes are a less well investigated class of compounds. Digermene **2**, being a rather unusual bicyclic example, was thus considered an interesting opportunity for the study of some basic spectroscopic and electrochemical properties[39, 40] of this compound class. Voltammograms of **2** at a glassy carbon (GC) electrode are shown in Figure 6. The first reduction peak (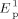
=−1.835 V) is both electrochemically and chemically reversible (at *v*=1 V s^−1^, Δ*E*_p−p/2_=64 mV and 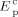
−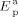
=58 mV, practically theoretical values for a fast electron transfer).[41] By comparison with the limiting current *i*_p_ of the reversible oxidation of ferrocene under similar conditions or by using the Cottrell slope from potentiostatic chronoamperometry, the process was shown to involve the transfer of one electron per molecule (*n*=1) corresponding to a **2/**[**2**]**^.−^** redox couple with the apparent standard potential *E*_o_(**2**/[**2**]**^.−^**)=−1.807 V. The first reduction step is followed by a second reduction at 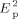
=−2.166 V (Δ*E*_p−p/2_=58 mV and 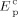
−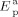
=60 mV) with the same electron stoichiometry, *n*=1. This electron transfer (ET) is also electrochemically reversible and corresponds to the [**2**]**^.−^**/[**2**]^**2−**^ redox pair. Both reduction peaks are diffusion controlled (*i*_p_/*v*^1/2^=constant for 0.2≤*v*≤10 V s^−1^). Remarkably, not only the injection of two electrons into the vacant orbitals of **2** does not provoke its irreversible chemical transformations but it also does not cause any substantial structural reorganization either in [**2**]**^.−^** or in [**2**]^**2−**^ that could be seen through the increased reorganization energy, *λ*, and decreased ET rate.[42]

In DME (a solvent of relatively low polarity, *ε*=7.07, *n*_D_=1.378),[43] the outer sphere reorganization energy, *λ*_o_, that is, the part of total reorganization energy that is related to redistribution of the solvent molecules accompanying the transfer of one electron to **2** is quite modest and amounts to ca. 19 kJ mol^−1^ in Marcus[44] or ca. 36 kJ mol^−1^ in Hush[45] approximations.^1^ Since at *v*=1 V s^−1^, overall reorganization energy (*λ*_i_+*λ*_o_) does not control ET rate yet, its rate constant, *k*_s_=[*FvD*/*RT*]^1/2^ (where *F* is the Faraday constant and *D* is the diffusion coefficient)[42] is expected to be greater than 0.014 cm s^−1^. With this value as a lower limit, a maximal contribution of internal reorganization energy, *λ*_i_, provoked by ET can be assessed from following equation: *k*_s_=*Z*_het_*κ*exp(−Δ*G*^≠^/*RT*), where *Z*_het_ is the heterogeneous collision frequency, for electrochemical reactions usually taken as 2×10^3^ cm s^−1^,[46] *κ* is the transmission coefficient, assumed to be unity for an adiabatic process, and Δ*G*^≠^ is the free energy of activation from Δ*G*^≠^=(*λ*/4)(1+*F*[*E*_p_−*E*^o^−*ϕ*]/*λ*)^2^, with *λ*=*λ*_i_+*λ*_o_.[44] Now, in order to assume *k*_s_>0.014 cm s^−1^, *λ* must be less than 113 kJ mol^−1^, that is, *λ*_i_<(113–36=77) kJ mol^−1^. In reality, both reduction peaks do not show any appreciable ET limitations up to *v*=10 V s^−1^, meaning that *λ*_i_ is even smaller, *λ*_i_<67 kJ mol^−1^.

The oxidation of **2** is characterized by one diffusion-controlled peak^2^ (*E*_p_=0.315 V), which also has electron stoichiometry of an electrochemically reversible process (*n*=1), in spite of its somewhat large half-width, Δ*E*_p−p/2_=95 mV (*v*=1 V s^−1^). When increasing the scan rate (*v*>10 V s^−1^), the cathodic branch on the voltammogram of the couple [**2**]**^.+^**/**2** starts to appear (Figure 6) but electron-transfer kinetics becomes limiting for the overall rate of oxidation, so the oxidation peak width, Δ*E*_p−p/2_, increases (300 mV at *v*=25 V s^−1^) with no substantial increase in the cathodic counterpart of the anodic peak.

**Figure 7:**
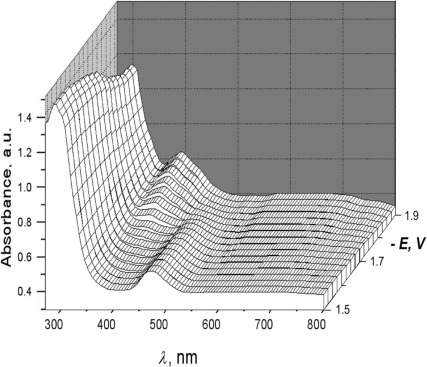
UV/Vis spectroelectrochemistry of the reduction of 2 in DME/0.1 m Bu_4_NPF_6_ at a Pt micro gauze working electrode; scan rate *v*=10 mV s^−1^, *T*=293 K. Front slice corresponds to UV/Vis spectrum of 2, back slice corresponds to the spectrum of electrogenerated anion radical [2]^.−^.

Reversibility of ET at the first reduction step allowed us to study the anion radical species [**2**]**^.−^** by real-time UV-spectroelectrochemistry. Corresponding cation radicals [**2**]**^.+^** are visibly less stable: even at 250 K, it was not possible to measure their UV spectrum. During the cathodic scan from −1.5 V to −1.9 V (*v*=10 mV s^−1^), the absorbance at *λ*=475 nm in the UV/Vis spectrum of **2** diminishes as the potential reaches the rising part of the polarization curve to totally disappear at *E*<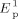. At the same time, the absorption of [**2**]**^.−^** electrogenerated at this reduction step appeared at *λ*=392 nm (Figure 7).

The absorbance maximum at *λ*=475 nm for neutral **2** corresponds to the HOMO–LUMO π(Ge=Ge)→π*(Ge=Ge) transition (see below). Taking into account that the potentials of reversible electrochemical processes relate to the energies of the corresponding frontier orbitals (under similar experimental conditions and supposing solvation free energies and working terms—the energies necessary to bring the corresponding species to the reaction zone—to be equal for both reduction and oxidation),[42] this value (ca. 2.6 eV) is somewhat higher than the apparent electrochemical hardness of this molecule, Δ*E*=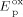
−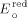
=2.12 V. However, the latter value is underestimated owing to two factors: the negative kinetic shift of 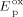 relative to 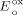, which might reach several hundred mV,[41] and the fact that in spite of a large size of **2**, solvation energies for charged radicals [**2**]**^.−^** and [**2**]**^.+^** are supposed to be greater than that of the neutral molecule, which diminishes both terms in the experimental Δ*E*. For these reasons and at the given 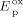 and 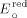, the band at *λ*=330 nm arises from a transition with energy higher than the HOMO–LUMO gap (π(Ge=Ge)→σ*(Si–Si), according to DFT calculations).

**Figure 8:**
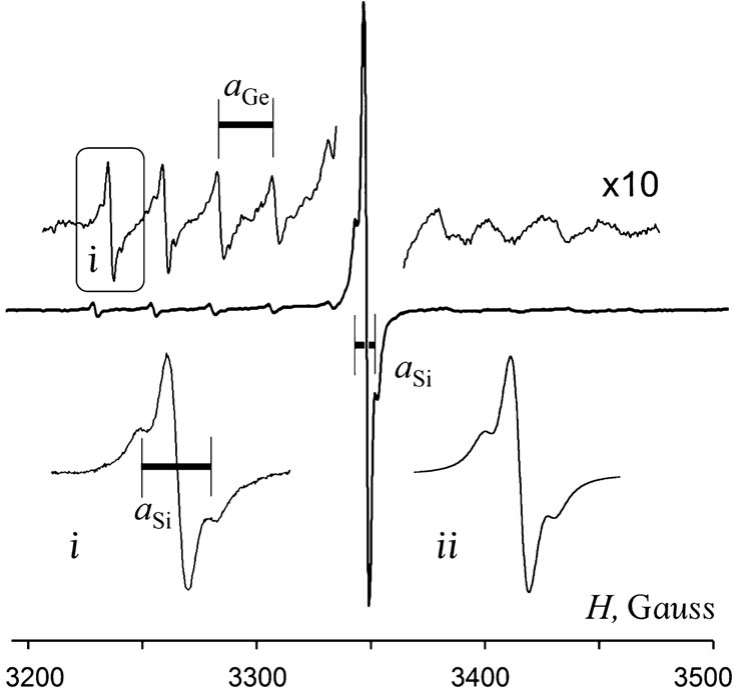
ESR spectrum of anion radicals [2]^.−^ electrogenerated at −1.7 V in DME/0.1 m Bu_4_N[B(C_6_F_5_)_4_] at a Pt micro spiral cathode. i) Low-field ^73^Ge satellite with two ^29^Si satellites; ii) simulated signal with two ^73^Ge and four ^29^Si ESR atoms.

Real-time ESR-coupled spectroelectrochemistry confirmed the paramagnetic character of the species produced by electroreduction of **2** at 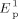. The central line in the spectrum of the radical anion (*g*=2.0272) is accompanied by ten ^73^Ge satellites with *a*_Ge_=22.09 G. The spectrum has well-resolved ends, which permits as well observing the satellites from ^29^Si (*a*_Si_=7.8 G), not only on the central line but also on all low-field ^73^Ge satellites (Figure 8). The integration of ^73^Ge satellites makes up ca. 15.9 % (twofold ^73^Ge natural abundance, 7.8 %) versus the central peak, while that of ^29^Si satellites corresponds to four Si atoms (4×4.67 %) indicating that the Ge–Ge linkage is not broken by the first electron uptake. The small value of the ^73^Ge hyperfine coupling constant (hfcc) *a*_Ge_ in [**2**]**^.−^** reflects the localization of unpaired electron mostly on the germanium p-type (p_z_) orbital that has a node at the nuclei. The adjacent Si atoms are located in the nodal plane and therefore have very small spin interaction with Ge. Although Sekiguchi et al.[50] reported very close hfcc values for ^73^Ge and ^29^Si (*a*_Ge_=20 G and *a*_Si_=7.3 G) for the planar (*t*Bu_2_MeSi)_3_Ge^.^ radical, the spin in [**2**]**^.−^** interacts with two Ge atoms implying that proper hfcc values are larger than the observed time-averaged values. This means that rapid exchange between spin- and charge-carrying Ge atoms of the Ge=Ge bridge (one closer to planar and another to pyramidal geometry, respectively), similar to that in the anion radical of acyclic disilene (*t*Bu_2_MeSi)_2_Si=Si(*t*Bu_2_MeSi)_2_**^.^**,[51] might occur in [**2**]**^.−^**. In this species, the Ge radical center shows slight pyramidality (even doubled, the experimental *a*_Ge_ value is still smaller than those reported for pyramidal R_3_Ge**^.^** radicals).[52]

Upon further increasing the cathodic potential (*E*_work_=−2.0→2.1 V), the signal of [**2**]**^.−^** vanished, corresponding to the transformation of this paramagnetic species into diamagnetic dianion [**2**]^2**−**^. This process is chemically reversible, since returning to E=−1.7 V (zone of the limiting current of the oxidation of [**2**]^2**−**^ back into [**2**]**^.−^**) made the spectrum of [**2**]**^.−^** reappear, in agreement with the data of cyclic voltammetry. Similar chemical reversibility of both reduction steps on the ESR scale was also observed for thiatetragermacyclopentene[53] and thiatetrasilacyclopentene[49] with endocyclic M=M bonds.

**Figure 9:**
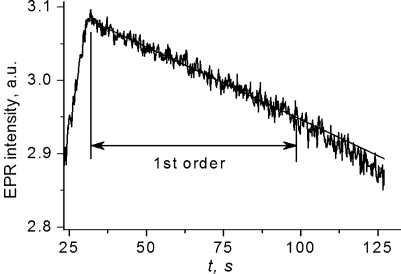
Decay of electrogenerated [2]^.−^ from fixed-field temperature-dependent ESR measurements. Static field *H*=3338.5 G, modulation amplitude *a*=2 G, other experimental conditions as in Figure 8.

Kinetics of the decay of anion radicals [**2**]**^.−^** (Figure 9) corresponds to a unimolecular process. With time, the formal kinetic order becomes smaller than unity probably because of signal broadening with a concomitant decrease in the peak intensity. The log(*k*)–*T* plot, obtained from temperature-dependent ESR measurements, permitted the determination of the apparent activation energy of this process, Δ*G*^≠^=17.2±3.2 kJ mol^−1^ and, through the Eyring equation (supposing *kT*/*h*=8.1×10^12^ s^−1^), its activation entropy, Δ*S*^≠^=−189 J mol^−1^ K^−1^.

With the rate constant *k*_298_=1.2×10^−3^ s^−1^, the Δ*G*^≠^ value features the anion radical as a relatively stable species. Activation entropy, Δ*S*^≠^, seems unusually high for a unimolecular reaction. Since large reorganization energy has not been revealed by voltammetry, this is not related to rehybridization of one Ge becoming substantially more tetrahedral relative to neutral **2**. One possible reason for the large Δ*S*^≠^ value might be a large release of steric strain energy (or twisting around the Ge=Ge bond)[54] in the reaction, accounting for the decay of [**2**]**^.−^**. Another reason might be that the decay of [**2**]**^.−^** starts with the population of a different electronic state of higher symmetry. Of course, overestimation of the probability factor in the Arrhenius equation would also contribute to a high Δ*S*^≠^ value.

**Figure 10:**
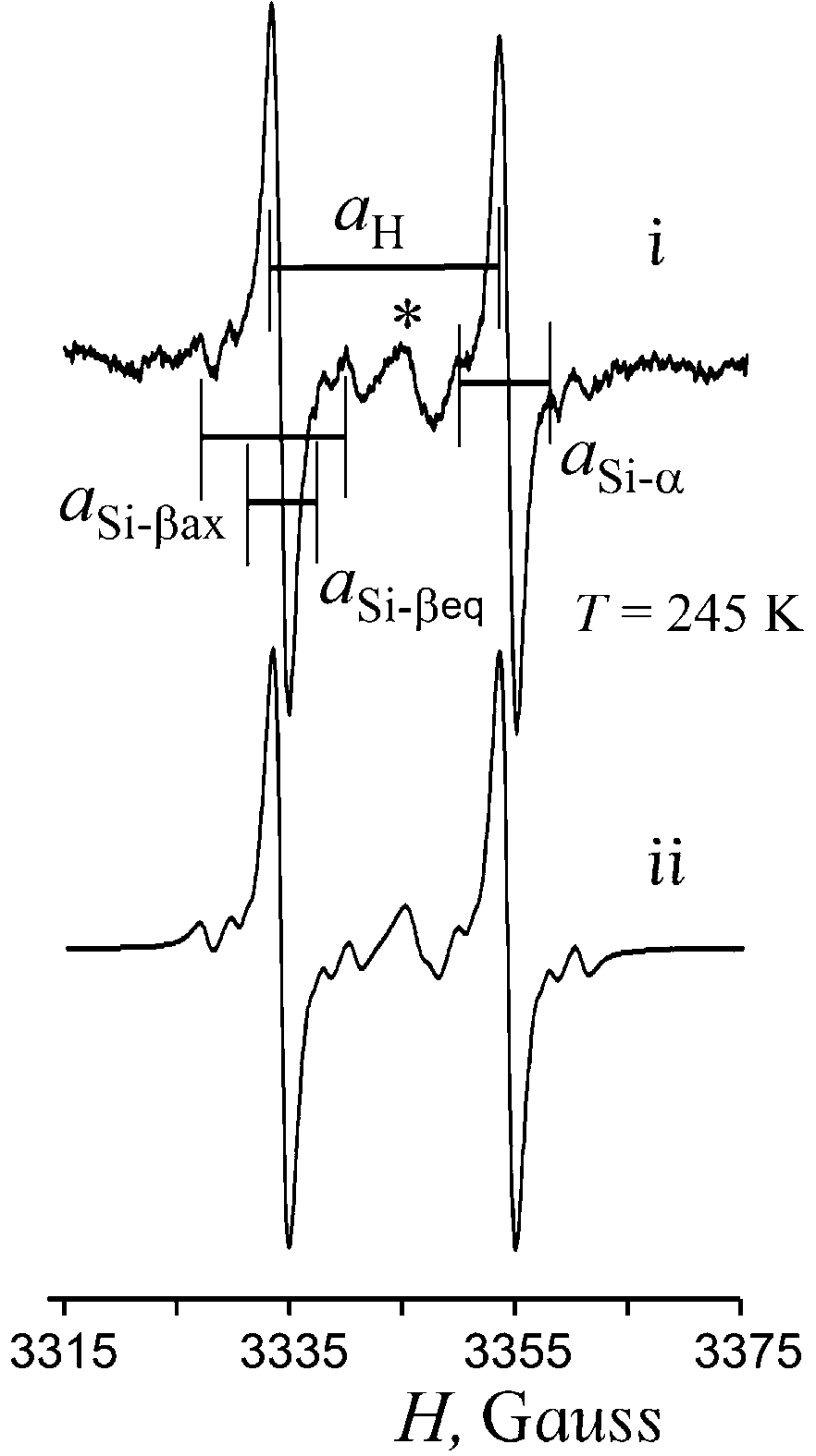
ESR spectrum of radical species electrogenerated from 2 at *E*=+0.3 V in DME/0.1 m Bu_4_N[B(C_6_F_5_)_4_] at a Pt anode. i) Experimental signal of [2]^.+^. ii) Simulated signal of [2]^.+^. (*) Residual signal of [2]^.+^.

The oxidation of **2**, set up in the ESR cell at the potential +0.3 V, produces cation radicals, [**2**]**^.+^**, which are less stable than anion radicals, [**2**]**^.−^** (Figure 6). The ESR signal of [**2**]**^.+^** (*g*=2.0238) could only be observed for 10 s at 245 K (central line, Figure 10). The decay of [**2**]**^.+^** results in secondary Ge-centered radical species (*g*=2.0258) that accounts for the emerging doublet-type spectrum (Figure 10). Its doublet pattern arises from the coupling with one nucleus of spin *I*=1/2 (for example, ^1^H or ^19^F) and hfcc value, *a*=19.98 G. Though ^73^Ge satellites (ten lines for ^73^Ge with *I*=9/2) of this doublet are lost in the noise, three groups of ^29^Si satellites (two lines for *I*=1/2) are well seen. Reconstruction of the spectrum (2*a*_Si-α_=7.95 G (8.4 %), 2*a*_Si-βax_=13.01 G (9.3 %) and 2*a*_Si-βeq_=5.37 G) features this species as containing planar Ge with two Si(SiMe_2_) branches and a broken Ge–Ge bond. Two Si atoms (Si(1) and Si(1′)) with *a*_Si-α_=7.95 G are located in the nodal plane of the spin-carrying p_z_(Ge) orbital (see Figure 3) so their small hfcc value is due to the interactions mostly by spin polarization. With respect to this orbital, two SiMe_3_ groups at these α-Si atoms are non-equivalent. The geometry of the Ge(Si(SiMe_3_)_2_)_2_ fragment is such that while one Si–SiMe_3_ σ-bond (β_ax_-Si atom) is almost parallel to p_z_(Ge), the second one forms a substantial dihedral angle with it, ∢p_z_-Ge-Si(1)-SiMe_3_ (for example, −53.92° in neutral **2**, Figure 3). With spin interactions of p-type orbitals being related to the cosine of this angle,[55] their hfcc values are assigned as *a*_Si-βax_=13.01 G and *a*_Si-βeq_=5.37 G (β-Si atoms denoted as β_ax_ and β_eq_ reflecting the orientation of the corresponding σ(Si–SiMe_3_) bonds). Atoms Si(2) and Si(2′), formally also at a β-position to Ge, have zero contribution because they are located in the plane perpendicular to the p_z_(Ge) orbital.

Since the interaction of [**2**]**^.+^** with the only possible external F-donor, the (C_6_F_5_)_4_B^−^ anion, is not very likely for steric reasons (also, the hfcc value of ^19^F is expected to be higher; for example, 65.21 G in Me_2_Si(F)**^.^**[56] and 31.9 G in *t*Bu_2_MeSi(F)**^.^**[57]), the doublet pattern presumably arises from a proton at Ge, resulting from intramolecular hydride transfer in the cation radical. With time, this radical evolves, giving rise to a non-identified C-centered organic radical (*g*=2.0026).

### Computational Study

**Figure 11:**
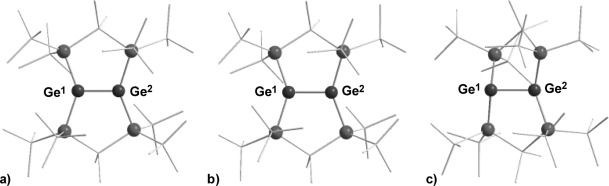
Ball and stick representation of the computed molecular structures of digermene 2 and its radical ions; the Si_2_Ge^1^–Ge^2^Si_2_ core is accentuated, the polysilane backbone is shown in the wireframe mode. a) 2; important parameter of the Ge–Ge linkage: Ge^1^–Ge^2^ 228.4 pm, Si-Ge^1^-Si 141.4°, Si-Ge^2^-Si 141.4°; bent angle *β*^1^ 5.8°, *β*^2^ 5.8°; twist angle *τ* 18.8°. b) [2]^.+^; Ge^1^–Ge^2^ 239.0 pm, Si-Ge^1^-Si 143.7°, Si-Ge^2^-Si 143.7°, bent angle *β*^1^ 1.9°, *β*^2^ 1.9°; twist angle *τ* 18.8°. c) [2]^.−^; Ge^1^–Ge^2^ 246.6 pm, Si-Ge^1^-Si 118.6°, Si-Ge^2^-Si 129.3°, bent angle *β*^1^ 92.5°, *β*^2^ 50.1°, twist angle *τ* 12.5°. Calculated at M062X/6-311+G(d,p) (Si,C,H) def2tzvp (Ge); color code: black (Ge), dark gray (Si), light gray (C), hydrogen atoms are omitted for clarity.

The results of density functional calculations were used to gain some further insights into the structure and bonding situation of the fleeting intermediates of the electrochemical processes, radical cation [**2**]**^.+^**, and radical anion [**2**]**^.−^**. The applied theoretical method[25] is justified by the good agreement between the experimental structural data obtained for bicyclic digermene **2** and that predicted by the computations. The calculated molecular structure of digermene **2** is, in all important details, very close to that determined by XRD. Even relatively weak modes, as for example, the *trans* bending or the twisting of the Ge=Ge bond are reproduced with high accuracy (*trans–*bent angle *β*: 2.5° (XRD) versus 5.8° (calcd); twist angle, *τ* : 16.2° (XRD) versus 18.8° (calcd), see Figure 3 and 11 and the Supporting Information, Table S2, for further details). In addition, the calculated UV data for digermene **2** can be used to gauge the quality of the calculated structure. The dominating bands in the UV spectra of digermene **2** at *λ*_1_=330 nm and at *λ*_2_=470 nm are assigned to the π(Ge=Ge)→ σ*(Si–Si) transition (*λ*_1_(calcd)=337 nm) and to the π(Ge=Ge)→π*(Ge=Ge) transition (*λ*_2_(calcd)=485 nm). A relative low ionization energy, IP, of digermene **2** to give corresponding radical cation [**2**]**^.+^** is predicted by the computations (IP=538 kJ mol^−1^,(5.57 eV))[58] in agreement with a high-lying HOMO of π(Ge=Ge) character (see the Supporting Information for surface plots of the frontier orbitals of digermene **2**). The removal of one electron from the π(Ge=Ge) orbital results in a significant elongation of the Ge=Ge bond (239.0 pm versus 228.4 pm in the digermene **2**, ca 5 % elongation). The flexible polysilane framework can, however, compensate for this pure bond elongation and the overall structure of digermene **2** is not changed significantly upon ionization (Figure 11).

**Figure 12:**
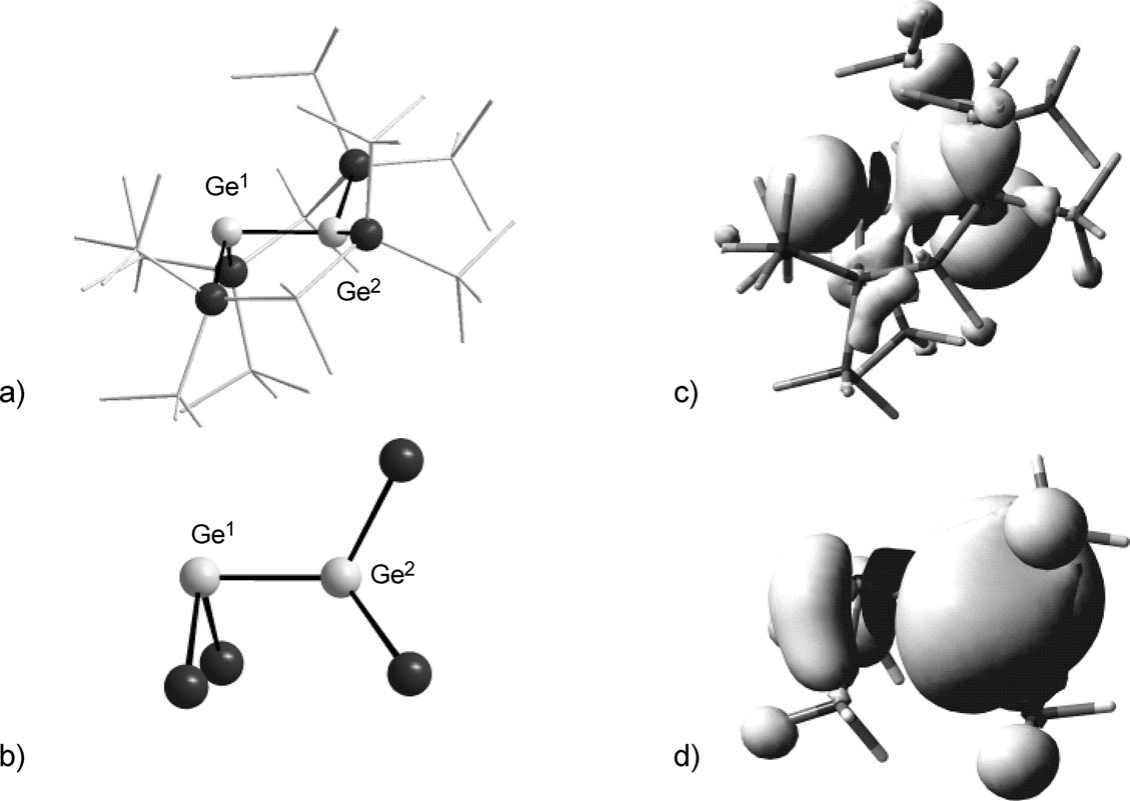
Ball and stick representation of the computed molecular structures of radical anions. a) [2]^.−^; the Si_2_Ge^1^–Ge^2^Si_2_ core is accentuated; the polysilane backbone is shown in the wireframe mode; important parameter of the Ge–Ge linkage: Ge^1^–Ge^2^ 246.6 pm, Si-Ge^1^-Si 118.6°, Si-Ge^2^-Si 129.3°, bent angle *β*^1^ 92.5°, *β*^2^ 50.1°, twist angle *τ* 12.5°. b) [25]^.−^ Ge^1^–Ge^2^ 250.4 pm, Si-Ge^1^-Si 90.4°, Si-Ge^2^-Si 104.6°, bent angle *β*^1^ 88.9°, *β*^2^ 39.4°, twist angle *τ* 73.9°. Calculated at M062X/6-311+G(d,p) (Si,C,H) def2tzvp (Ge); color code: light gray (Ge), black (Si), gray (C), hydrogen atoms are omitted for clarity. c) Calculated SCF spin density (difference between α and β spins) of radical anion [2]^.−^. d) Calculated SCF spin density (difference between α and β spins) of radical anion [25]^.−^. Positive spin density (white), negative spin density (black), surface isodensity value 0.0008, color code: light gray (Ge), black (Si), gray (C), white (H). Calculated at B3LYP/def2tzvp//M062X/6-311+G(d,p) (Si,C,H); def2tzvp(Ge)).

The electron affinity of digermene **2** to give the corresponding radical anion is substantial (Δ*E*_A_=−128 kJ mol^−1^). The structural consequences of the one-electron reduction are also remarkable: the Ge=Ge bond is markedly stretched (246.6 pm versus 228.4 pm in the digermene **2**, ca. 8 % elongation) and both germanium centers are significantly pyramidalized, although to a different extent (bent angle *β*(Ge^1^)=92.5° and *β*(Ge^2^)=50.1°; see Figure 12 a). The SOMO of radical anion [**2**]**^.−^** is of π* symmetry and it is delocalized over both germanium atoms (see Figure S3 in the Supporting Information). Due to the non-symmetrical structure of radical anion [**2**]**^.−^**, its calculated spin density (Figure 12 b) is different at both germanium atoms.

This result suggests that the observed equivalence of the two germanium atoms by ESR spectroscopy is only time averaged. The SOMO of radical anion [**2**]**^.−^** has almost pure π*(Ge=Ge) character (see the Supporting Information for a surface diagram of the SOMO), with nodal planes at the germanium atoms. Therefore only small hyperfine interactions between the unpaired electron and the germanium atoms are to be expected, in qualitative agreement with the experimental observation.

The related acyclic persilylated disilene and distannene radical anions, [**23**]**^.−^** and [**24**]**^.−^** show charge/radical separation in the solid state and in solution.[37, 51, 59] In the case of disilene radical anion [**23**]**^.−^**, an equilibrium between equivalent structures that is fast on the ESR timescale exhibits a symmetric structure at room temperature.[51]


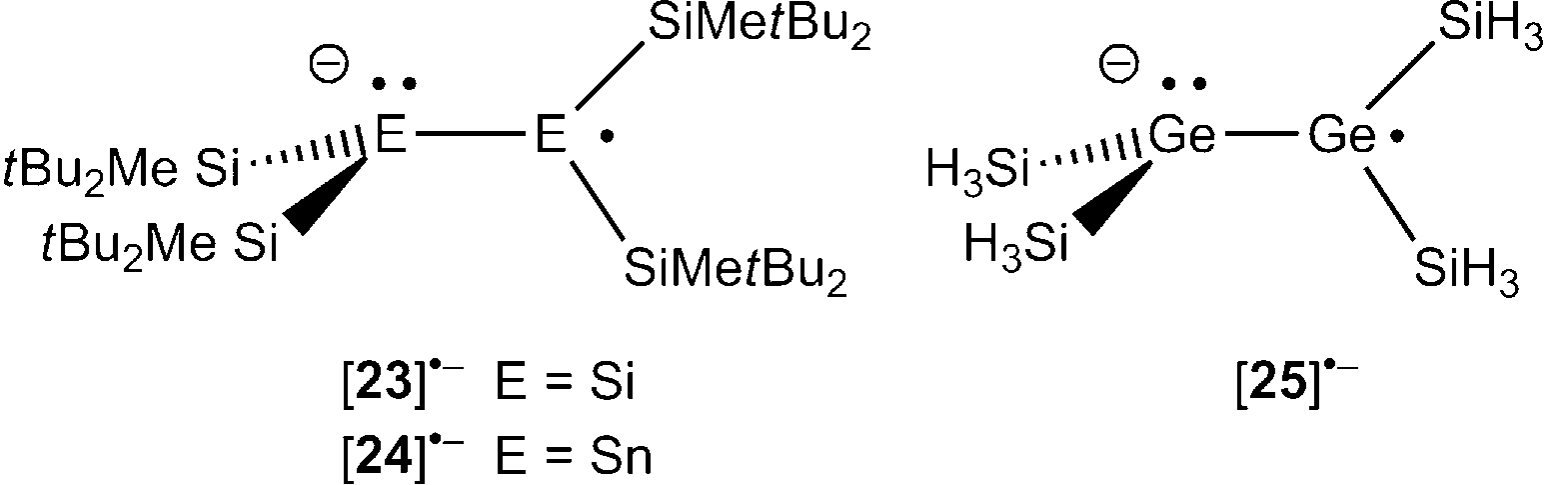


The solid-state structures of both radical anions showed a strong twist around the E–E bond. In addition, one tetrel atom E was found significantly pyramidalized, indicating the localization of an electron lone pair at this position. The second tetrel atom showed an almost planar coordination, which is typical for persilylated tetryl radicals. The results of structure optimizations for acyclic persilylated digermenyl radical anion [**25**]**^.−^** predict also for this model compound radical/charge separation. The germanium center, Ge^1^, of radical anion [**25**]**^.−^** is strongly pyramidalized (*β*(Ge^1^)=88.9°) while at the less pyramidalized germanium atom, Ge^2^, (*β*(Ge^2^)=39.4°) an extremely high spin density is predicted. (Figure 12 b, d). The strong twist of the molecule around the Ge^1^–Ge^2^ bond efficiently separates the radical and anion parts (*τ*=73.9°). The comparison between the calculated structures of model radical anion [**25**]**^.−^** and [**2**]**^.−^** indicates also for the latter the onset of the separation between the anion and the radical. This is shown by the strong pyramidalization of germanium atom Ge^1^ and by the significant higher spin density at the second atom, Ge^2^ (Figure 12 a, c). The fused bicyclic topology of radical anion [**2**]**^.−^** obviously prevents the highly twisted arrangement of Ge–Ge bond, which is needed for a complete separation and allows for a certain delocalization of the additional electron across both germanium atoms, as indicated by the calculated spin density for radical anion [**2**]**^.−^** (Figure 12 c).

## Conclusion

The chemistry of silylated tetrylenes and the respective compounds containing double bonds between higher Group 14 elements has made remarkable progress in recent years.[2] However, while a fair number of compounds of this type now exists, reactivity and properties of these compounds are still not fully understood.

The current account concentrates on the synthesis of base-stabilized disilylated germylenes and the subsequent reaction of these to give digermenes. Digermene formation is critically dependent on the stabilization of the intermediate germylene by a phosphane base. Base-adduct formation allows the use of comparably small silyl substituents. In the absence of the base under the reaction conditions, further reaction of germylene with a silanide would occur. The choice of phosphanes as bases compared to N-heterocyclic carbenes is also important as phosphanes can easily be removed in order to release a free germylene, which can undergo dimerization or rearrangement reactions.

Recently we reported on the formation of a five-membered germylene adduct, which after releasing the free germylene, underwent a 1,2-silyl shift to form a silagermene and subsequently dimerized by way of a [2+2] cycloaddition.[20] The tetrasilylated bicyclic digermene **2**, described in the present study, is formed from a related four-membered germylene adduct. A closer examination of the different reaction pathways revealed a much higher degree of stabilization of the four-membered germylene dimer (that is, respective digermene **11**) compared to its five-membered counterpart, **14**, as the decisive factor for the different behavior.

Obtained bicyclic digermene **2** was subjected to a voltammetric study, which revealed two reversible reduction waves leading to respective radical anion [**2**]**^.−^** and further to dianion [**2**]^**2−**^. The formation of [**2**]**^.−^** was further substantiated by ESR and UV spectroscopy. In contrast to the fairly stable reduction products, radical cation [**2**]**^.+^**, formed by one-electron oxidation, was found not to be stable enough to be studied by ESR spectroscopy. As chemical one-electron oxidation of a tetrasilyldisilene by Ph_3_C^+^⋅B(C_6_F_5_)_4_^−^ was reported to yield a stable isolable radical cation,[36] the reason for the low stability of [**2**]**^.+^** is likely its bicyclic nature. Presumably, initially formed [**2**]**^.+^** decomposed by an intramolecular hydride shift reaction. This was suggested by the observed ESR spectrum, which featured a germyl radical with strong coupling to a hydrogen atom.

By means of theoretical calculations, the structure of digermene **2** and radical anion [**2**]**^.−^** were studied and the structure and spectroscopic features of digermene **2** were found in good agreement with experimental observations. For radical anion [**2**]**^.−^**, the onset of radical/charge separation was suggested by the computed structural parameter and by the predicted spin distribution. Structural restrictions induced by the fused bicyclic structure of digermene radical anion [**2**]**^.−^** prevent the perfected radical/charge separation as it is reported for related disilene and distannene radical anions.[37, 51, 59] Nevertheless, the computational results suggest that the electronic situation in digermene radical anion [**2**]**^.−^** is clearly different from that of alkene radical anions with a planar C–C core and symmetrically distributed spin density.[60]

## Experimental Section

### General remarks

All reactions involving air-sensitive compounds were carried out under an atmosphere of dry nitrogen or argon using either Schlenk techniques or a glove box. All solvents were dried using a column-based solvent purification system.[61] Potassium *tert*-butanolate was purchased from Merck. All other chemicals were obtained from different suppliers and used without further purification. ^1^H (300 MHz), ^13^C (75.4 MHz), and ^29^Si (59.3 MHz) NMR spectra were recorded on a Varian INOVA 300 spectrometer. If not noted otherwise, for all samples, C_6_D_6_ was used as solvent. To compensate for the low isotopic abundance of ^29^Si, the INEPT pulse sequence[62, 63] was used for the amplification of the signal. NMR reaction-control measurements were done by analyzing aliquots, without work-up, by adding a D_2_O capillary to provide a lock signal.

### 1,5-Digerma-2,2,4,4,6,6,8,8-octakis(trimethylsilyl)bicyclo[3.3.0]octasil-9-ene (2)

A solution of GeBr_2_^.^dioxane (705 mg, 2.20 mmol) and trimethylphosphane (167 mg, 2.20 mmol) in THF (10 mL) was cooled to −60 °C and **1**[21] in DME (10 mL) was added dropwise. The reaction was stirred for 3 h at −60 °C unless NMR control measurements showed complete conversion to the PMe_3_ adduct **3** (^29^Si NMR (D_2_O capillary): *δ*=−7.7 (d, ^3^*J*_Si,P_=19 Hz, SiMe_3_), −10.1 (d, ^3^*J*_Si,P_=15 Hz, SiMe_3_), −106.3 ppm (d, ^2^*J*_Si,P_=18 Hz, Si_q_); ^31^P NMR (D_2_O capillary): *δ*=−19.1 ppm). After warming the reaction mixture up to −20 °C, the solvent was removed and a dark red residue remained, which was treated three times with pentane. The pentane layers were reduced to 5 mL and upon storage at −20 °C, compound **2** was obtained as orange needles (327 mg, 17 %). M.p. 347 °C (dec); ^1^H NMR (300 MHz): *δ*=0.71 (s, 12 H, Me_2_Si), 0.40 ppm (s, 72 H, Me_3_Si); ^13^C NMR (75.4 MHz): *δ*=3.9 (Me_2_Si), 3.5 ppm (Me_3_Si); ^29^Si NMR (59.3 MHz): *δ*=−9.7 (Me_3_Si), −10.5 (Me_2_Si), −92.7 ppm (Si_q_); UV/Vis (pentane): *λ*_1_=330 nm (*ε*_1_=7.6×10^3^ mol^−1^ dm^3^ cm^−1^), *λ*_2_=470 nm (*ε*_2_=1.3×10^4^ mol^−1^ dm^3^ cm^−1^); elemental analysis calcd (%) for for C_28_H_84_Ge_2_Si_14_ (959.43): C 35.05, H 8.83; found: C 35.86, H 7.82.

## Footnotes

1 Bulkiness of **2**, implying that the site of ET is relatively far from the electrode so that electrode-image effects can be neglected, acts in favor of Hush reorganization energy;[45] however, ET is still supposed to be adiabatic.[42]

2 Putting aside the uncertainty in the potentials of reference electrodes and non-homogeneity of the experimental electrochemical data of heavy alkenes, one can see that their oxidation generally occurs at 0.3–0.5 V versus SCE, though both higher and lower values have been reported.[8, 40, 47–49] The published reduction potentials cover a larger span, from −0.46 to −2.66 V, probably implying that they need to be carefully revised.

## Supporting Information

As a service to our authors and readers, this journal provides supporting information supplied by the authors. Such materials are peer reviewed and may be re-organized for online delivery, but are not copy-edited or typeset. Technical support issues arising from supporting information (other than missing files) should be addressed to the authors.

miscellaneous_information

## References

[1] Mizuhata Y, Sasamori T, Tokitoh N (2009). Chem. Rev.

[2] Lee VY, Sekiguchi A (2010). Organometallic Compounds of Low-Coordinate Si, Ge, Sn and Pb: From Phantom Species to Stable Compounds.

[3] Hahn FE, Zabula AV, Pape T, Hepp A, Tonner R, Haunschild R, Frenking G (2008). Chem. Eur. J.

[4] Blom B, Stoelzel M, Driess M (2013). Chem. Eur. J.

[5] Asay M, Jones C, Driess M (2011). Chem. Rev.

[6] Matsuo T, Kobayashi M, Tamao K (2010). Dalton Trans.

[7] Kira M (2004). J. Organomet. Chem.

[8] Kira M, Iwamoto T (2000). J. Organomet. Chem.

[9] Okazaki R, West R (1996). Adv. Organomet. Chem.

[10] Power PP, Lattman M, Kemp RA (2005). Modern Aspects of Main Group Chemistry: ACS Symposium Series Vol. 917.

[11] Tokitoh N, Okazaki R, Rappoport Z (2002). The Chemistry of Organic Germanium, Tin and Lead Compounds.

[12] Weidenbruch M, Rappoport Z, Apeloig Y (2001). The Chemistry of Organic Silicon Compounds.

[13] Weidenbruch M (2002). J. Organomet. Chem.

[14] Weidenbruch M (1999). Eur. J. Inorg. Chem.

[15] Petz W (1986). Chem. Rev.

[16] Lappert MF, Rowe RS (1990). Coord. Chem. Rev.

[17] Leung W-P, Kan K-W, Chong K-H (2007). Coord. Chem. Rev.

[18] Harris DH, Lappert MF (1974). J. Chem. Soc. Chem. Commun.

[19] Rekken BD, Brown TM, Fettinger JC, Tuononen HM, Power PP (2012). J. Am. Chem. Soc.

[20] Hlina J, Baumgartner J, Marschner C, Albers L, Müller T (2013). Organometallics.

[21] Fischer R, Frank D, Gaderbauer W, Kayser C, Mechtler C, Baumgartner J, Marschner C (2003). Organometallics.

[22] Kobayashi H, Iwamoto T, Kira M (2005). J. Am. Chem. Soc.

[23] Iwamoto T, Furiya Y, Kobayashi H, Isobe H, Kira M (2010). Organometallics.

[24] Hlina J, Baumgartner J, Marschner C, Zark P, Müller T (2013). Organometallics.

[27] Carter EA, Goddard WA (1986). J. Phys. Chem.

[28] Trinquier G, Malrieu JP (1987). J. Am. Chem. Soc.

[29] Malrieu JP, Trinquier G (1989). J. Am. Chem. Soc.

[30] Kira M, Iwamoto T, Maruyama T, Kabuto C, Sakurai H (1996). Organometallics.

[32] Jacobsen H, Ziegler T (1994). J. Am. Chem. Soc.

[33] Fischer R, Konopa T, Ully S, Baumgartner J, Marschner C (2003). J. Organomet. Chem.

[34] Lee VY, Takanashi K, Ichinohe M, Sekiguchi A (2003). J. Am. Chem. Soc.

[35] Inoue S, Ichinohe M, Sekiguchi A (2007). J. Am. Chem. Soc.

[36] Inoue S, Ichinohe M, Sekiguchi A (2008). J. Am. Chem. Soc.

[37] Fukawa T, Lee VY, Nakamoto M, Sekiguchi A (2004). J. Am. Chem. Soc.

[38] 10.1002/hc.21165.

[39] Sasamori T, Miyamoto H, Sakai H, Furukawa Y, Tokitoh N (2012). Organometallics.

[40] Schäfer A, Weidenbruch M, Müller T, Pravinkumar K, Becker JY (2009). Chem. Eur. J.

[41] Hammerich O, Hammerich O, Lund H (2000). Organic Electrochemistry.

[42] Savéant J-M (2006). Elements of Molecular and Biomolecular Electrochemistry: An Electrochemical Approach to Electron Transfer Chemistry.

[43] Karapetyan YA, Eychis VN (1989). Physico-chemical Properties of Non-aqueous Solutions of Electrolytes.

[44] Marcus RA (1957). J. Chem. Phys.

[45] Hush NS (1999). J. Electroanal. Chem.

[46] Amatore C, Hammerich O, Lund H (2000). Organic Electrochemistry.

[47] Bendikov M, Kravchenko V, Apeloig Y, Becker JY (2004). Organometallics.

[48] Shepherd BD, West R (1988). Chem. Lett.

[49] Zeitouny J, Jouikov V (2009). Phys. Chem. Chem. Phys.

[50] Sekiguchi A, Fukawa T, Nakamoto M, Lee VY, Ichinohe M (2002). J. Am. Chem. Soc.

[51] Sekiguchi A, Inoue S, Ichinohe M, Arai Y (2004). J. Am. Chem. Soc.

[52] Iley J, Patai S, Rappoport Z (1995). The Chemistry of Organic Germanium, Tin, and Lead Compounds, The Chemistry of Functional Groups, Vol 1.

[53] Jouikov VV (2008). ECS Trans.

[54] Kira M (2011). Organometallics.

[55] Gerson F, Huber W (2003). Electron Spin Resonance Spectroscopy of Organic Radicals.

[56] Merritt MV, Fessenden RW (1972). J. Chem. Phys.

[57] Sheberla D, Tumanskii B, Bravo-Zhivotovskii D, Molev G, Molev V, Lee VY, Takanashi K, Sekiguchi A, Apeloig Y (2010). Organometallics.

[58] http://webbook.nist.gov.

[59] Lee VY, Fukawa T, Nakamoto M, Sekiguchi A, Tumanskii BL, Karni M, Apeloig Y (2006). J. Am. Chem. Soc.

[60] Zheludev A, Grand A, Ressouche E, Schweizer J, Morin BG, Epstein AJ, Dixon DA, Miller JS (1994). J. Am. Chem. Soc.

[61] Pangborn AB, Giardello MA, Grubbs RH, Rosen RK, Timmers FJ (1996). Organometallics.

[62] Morris GA, Freeman R (1979). J. Am. Chem. Soc.

[63] Helmer BJ, West R (1982). Organometallics.

