# Supplementary material for: Formation and Properties of a Bicyclic Silylated Digermene
Source: Chemistry. 2014 Jun 30;20(30):9357–66. doi: 10.1002/chem.201402785 (PMC4506559; doi:10.1002/chem.201402785)
Supplement: Supplementary file 1 — miscellaneous_information [file chem0020-9357-sd1.pdf]

CHEMISTRY
A European Journal

Supporting Information

© Copyright Wiley-VCH Verlag GmbH & Co. KGaA, 69451 Weinheim, 2014

Formation and Properties of a Bicyclic Silylated Digermene

Johann Hlina,[a] Judith Baumgartner,*[b] Christoph Marschner,*[a] Lena Albers,[c] Thomas Müller,*[c] and Viatcheslav V. Jouikov*[d]

chem_201402785_sm_miscellaneous_information.pdf

Table of Contents

1.	Experimental Section	S2
1.1	Crystallographic Details (Table S-1)	S5
2.	Computational Details	S6
2.1	General	S6
2.2	Cartesian Coordinates (Table S-4)	S10
3.	References	S24

1. EXPERIMENTAL SECTION

General Remarks. All reactions involving air-sensitive compounds were carried out under an atmosphere of dry nitrogen or argon using either Schlenk techniques or a glove box. All solvents were dried using column based solvent purification system.[1] Potassium *tert*-butanolate was purchased from MERCK. All other chemical were obtained from different suppliers and used without further purification.

^{1}H (300 MHz), ^{13}C (75.4 MHz), and ^{29}Si (59.3 MHz) NMR spectra were recorded on a Varian INOVA 300 spectrometer. If not noted otherwise for all samples C_6D_6 was used as solvent. To compensate for the low isotopic abundance of ^{29}Si the INEPT pulse sequence[2,3] was used for the amplification of the signal. NMR reaction control measurements were done with aliquot samples without work up by adding a D_2O capillary providing a lock signal.

X-Ray Structure Determination. For X-ray structure diffraction analyses the crystals were mounted onto the tip of glass fibers, and data collection was performed with a BRUKER-AXS SMART APEX CCD diffractometer using graphite-monochromated Mo Kα radiation (0.71073 Å). The data were reduced to F^2_o and corrected for absorption effects with SAINT[4] and SADABS,[5,6] respectively. The structures were solved by direct methods and refined by full-matrix least-squares method (SHELXL97).[7] If not noted otherwise all non-hydrogen atoms were refined with anisotropic displacement parameters. All hydrogen atoms were located in calculated positions to correspond to standard bond lengths and angles. All diagrams were drawn with 30% probability thermal ellipsoids and all hydrogen atoms were omitted for clarity.

Crystallographic data (excluding structure factors) for the structures of compounds **2**, **21**, and **22** reported in this paper have been deposited with the Cambridge Crystallographic Data Center as supplementary publication no. CCDC-883864 (**2**), 883862 (**21**), and 883865 (**22**). Copies of data can be obtained free of charge at: http://www.ccdc.cam.ac.uk/products/csd/request/.

Voltammetry and spectroelectrochemistry. PAR 2373 and EG&G 273 potentiostats were used for cyclic voltammetry and for spectroelectrochemical experiments, respectively. A 5-mL conventional voltammetric three-electrode electrochemical cell and a three-electrode EPR spectroelectrochemical cell[13] were filled/operated in the glovebox. The measured potentials were corrected using E^0 of the reversible couple Fc^+/Fc (0.31 V vs. SCE)[14]. Supporting salts, $Bu_4N[B(C_6F_5)_4]$ and Bu_4NPF_6, were kept over P_2O_5 and activated overnight in vacuum at 80 °C

before being introduced into the glovebox. Dimethoxyethane was distilled from sodium benzophenone ketyl and kept over molecular sieves; once in the glovebox, it was additionally dried over 3A molecular sieves (activated in turbomolecular pump vacuum at 300 °C) for ~20 min before the experiments. EPR spectra were recorded at Bruker EMX X-band spectrometer (9.46 GHz), UV-Vis spectra were recorded in a 1 mm three-electrode quartz cell using a diode array Shimadzu Multispec-1500 spectrometer.

$GeBr_2$·dioxane,[8] 1,1,3,3-tetrakis(trimethylsilyl)dimethyltrisilanyl 1,3-dipotassium (**1**),[9] 2-germa-1,1,3,3-tetrakis(trimethylsilyl)-4,4-dimethylcyclotetrasilan-2-ylen·$ImMe_4$ (**5**),[10] 1,1,4,4-tetrakis(trimethylsilyl)octamethylcyclohexasilane 1,4-dipotassium·(18-crown-6)$_2$ (**19**),[11] and $ImMe_4$[12] were prepared according to literature procedures.

Compounds **2**, **21**, and **22** can also be prepared using $GeCl_2$·dioxane instead of $GeBr_2$·dioxane in comparable yields.

Tetracyclic digermene (21): A solution of $GeBr_2$·dioxane (769 mg, 2.40 mmol) and triethylphosphine (282 mg, 2.40 mmol) in THF (7 mL) and DME (7 mL) was cooled to -60 °C and a solution of **19** (2 mmol) in toluene (10 mL) was slowly added dropwise. The reaction was stirred for 3 h at -60 °C unless NMR control measurements showed the complete conversion to the PEt_3 adduct **20**. After warming up to rt the solvent was removed and a orange red residue remained which was treated three times with pentane/toluene (1:1 mixture). Removing of the solvents allowed the spectroscopic characterization of **20**: ^{1}H NMR (δ ppm): 1.40 (m, 6H, CH_2P), 0.79 (m, 9H, CH_3CH_2), 0.79 (m, 6H, Me_2Si), 0.49 (s, 6H, Me_2Si), 0.42 (s, 6H, Me_2Si), 0.40 (s, 6H, Me_2Si), 0.36 (s, 18H, Me_3Si), 0.32 (s, 6H, Me_2Si). ^{29}Si NMR (δ ppm): -6.4 (s, Me_3Si), -28.7 (d, $^3J_{Si,P}$ = 6.0 Hz, Me_2Si), -33.1 (d, $^3J_{Si,P}$ = 4.7 Hz, Me_2Si), -105.3 (d, $^2J_{Si,P}$ = 14.8 Hz, Si_q). ^{31}P NMR (δ ppm): 11.3. Crystallization of **20** at rt with pentane/toluene (1:1 mixture) afforded **21** as an orange solid (125 mg, 10 %). Mp: 343-347 °C (dec.). ^{1}H NMR (δ ppm): 0.49 (s, 24H, Me_2Si), 0.45 (s, 36H, Me_3Si), 0.39 (s, 24H, Me_2Si). ^{13}C NMR (δ ppm): 4.7 (Me_3Si), -0.2 (Me_2Si), -0.3 (Me_2Si). ^{29}Si NMR (δ ppm): -6.2 (Me_3Si), -41.0 (Me_2Si), -83.9 (Si_q). Anal. Calcd for $C_{28}H_{84}Ge_2Si_{16}$ (1015.60): C 33.11, H 8.34. Found: C 30.46, H 7.90.

7-Germa-1,4-bis(trimethylsilyl)-7-octamethylbicyclo[2.2.1]heptasilan-7-ylidene·$ImMe_4$ (**22**): A solution of **19** (344 mg) in DME (2.5 mL) / THF (2.5 mL) was added at -30 °C to a stirred mixture of $GeBr_2$·dioxane (121 mg, 0.38 mmol) and 1,3,4,5-tetramethylimidazol-2-ylidene (47

mg, 0.38 mmol) in THF (5 mL) within 5 min. The orange-red reaction mixture was stirred for 14 h at -30 °C. The solvent was removed and an orange-red residue remained which was treated three times with pentane. The pentane layers were reduced and crystallization by addition of toluene at -30 °C gave compound **22** as orange crystals (116 mg, 53%). Mp: 189-192 °C (dec.). ^{1}H NMR (δ ppm): 3.41 (s, 6H, MeN), 1.25 (s, 6H, MeC), 0.64 (s, 6H, Me_2Si), 0.53 (s, 12H, Me_2Si), 0.45 (s, 18H, Me_3Si), 0.13 (s, 6H, Me_2Si). ^{13}C NMR (δ ppm): 175.6 (NCN), 125.2 (Me*C*), 36.0 (*Me*N), 32.1 (*Me*N), 8.4 (*Me*C), 4.3 (Me_3Si), -0.6 (Me_2Si), -2.1 (Me_2Si). ^{29}Si NMR (δ ppm): -6.0 (Me_3Si), -29.6 (Me_2Si), -34.3 (Me_2Si), -105.7 (Si_q). Anal. Calcd for $C_{21}H_{54}GeN_2Si_8$ (631.99): C 39.91, H 8.61. Found: C 39.40, H 8.55.

Table S-1. Crystallographic data for compounds **2**, **21**, and **22**

	**2**	**21**	**22**
Empirical formula	$Ge_2Si_{14}C_{28}H_{84}$	$Ge_2Si_{16}C_{28}H_{84}$	$GeN_2Si_8C_{21}H_{54}$
M_w	959.39	1015.57	631.97
Temperature [K]	100(2)	100(2)	100(2)
Size [mm]	0.44x0.28x0.26	0.40x0.33x0.24	0.45x0.45x0.45
Crystal system	monoclinic	monoclinic	tetragonal
Space group	C2/c	P2(1)/c	P4(3)2(1)2
a [Å]	23.456(5)	17.412(3)	13.814(2)
b [Å]	9.880(2)	14.126(3)	13.814(2)
c [Å]	24.839(5)	19.437(4)	37.337(8)
α [°] = γ [°]	90	90	90
β [°]	105.36(3)	101.65(3)	90
V [$Å^3$]	5551(2)	5830(2)	7125(2)
Z	4	4	8
ρ_{calc} [gcm^{-3}]	1.148	1.157	1.178
Absorption coefficient [mm^{-1}]	1.403	1.379	1.142
F(000)	2048	2160	2704
θ range	$1.70<\theta<26.38$	$1.68<\theta<26.33$	$1.57<\theta<26.38$
Reflections collected/unique	19383/5654	45659/11836	57417/7302
Completeness to θ [%]	9.5	99.8	100
Data/restraints/parameters	5654/7/257	11836/0/443	7302/0/308
Goodness of fit on F^2	1.14	0.73	1.04
Final R indices [$I>2\sigma(I)$]	R1=0.063, *w*R2=0.154	R1=0.032, *w*R2=0.083	R1=0.026, *w*R2=0.063
R indices (all data)	R1=0.074, *w*R2=0.159	R1=0.041, *w*R2=0.090	R1=0.028, *w*R2=0.064
Largest diff. Peak/hole [e^-/ $Å^3$]	1.57/-0.37	0.86/-0.27	0.53/-0.20

2. Computational Details

2.1 General

All quantum chemical calculations were carried out using the Gaussian09 package.[15] The molecular structure optimizations were performed at the density functional M06-2X level of theory[16] using the def2-tzvp basis set for Ge and the 6-311+G(d,p) basis set for P, Si, C, H. Every stationary point was identified by a subsequent frequency calculation either as minimum (Number of imaginary frequencies (NIMAG): 0) or transition state (NIMAG: 1). The SCF energies, E(SCF), for all optimized molecular structures obtained with this method are given in Table S-2. Harmonic frequencies were calculated analytically and were used with standard scaling (0.893) to obtain corrections for enthalpy and entropy. The computed Gibbs free energies at T=298.15K and p=0.101 MPa (1 atm) in the gas phase, G^{298}, are also given in Table S-2. Intrinsic reaction coordinate (IRC) calculations[17,18] were used to connect transition state structures with the appropriate molecular structures of intermediates. The corresponding computed molecular structures are given in the form of their Cartesian coordinates in Table S-4. Exited state calculations have been done using the Time Dependent (TD) DFT method[19,20]using the B3LYP functional and a def2-tzvp basis[21] for all atoms. Calculated UV spectra for the gas phase were drawn by broadening of the individual excitations with gaussians with a bandwith of 0.1 eV (See Figure S-1). Relevant surface diagrams of molecular orbitals and spin densities were calculated from B3LYP/def2-tzvp densities (See Figures S-2 - S-4). Ionization potential (I_P) and electron affinities (E_A) were calculated using the B3LYP/def2-tzvp method and molecular structures obtained at the M06-2X/def2tzvp(Ge),6-311+G(d,p)(Si,C,H) level. Pertinent structural parameter of compounds **1**, **11** and **14** are summarized in Table S-3.

The following atomic units have been used: Energy E: 1 au = e^2 a_0^{-1} = 2625.498 kJ mol^{-1}

Table S-2. Absolute SCF energies E(SCF) and free Gibbs enthalpies at 298K , G^{298}, for compounds of interest (at M06-2X/def2tzvp (Ge), 6-311+G(d,p) (Si,C,H)

Compound	PG	E(SCF) [au/particle]	G^{298} [au/particle]	(Nimag) ν(Nimag) [cm^{-1}]
**4**	C_1	-4662.20719	-4661.75857	0
**TS(4/10)**	C_1	-4662.18581	-4661.73414	(1) -65.63
**10**	C_1	-4662.20489	-4661.75784	0
**11**	C_i	-9324.49569	-9323.55692	0
**12**	C_1	-9324.50139	-9323.55302	0

**2**	C_1	-9324.52326	-9323.58672	0
**[2]**$^{+\cdot}$	C_1	-9324.31861	-9323.37611	0
**[2]**$^{-\cdot}$	C_1	-9324.57986	-9323.64305	0
**3**	C_1	-5123.29722	-5122.73469	0
**PMe_3**	C_1	-461.04718	-460.96278	0

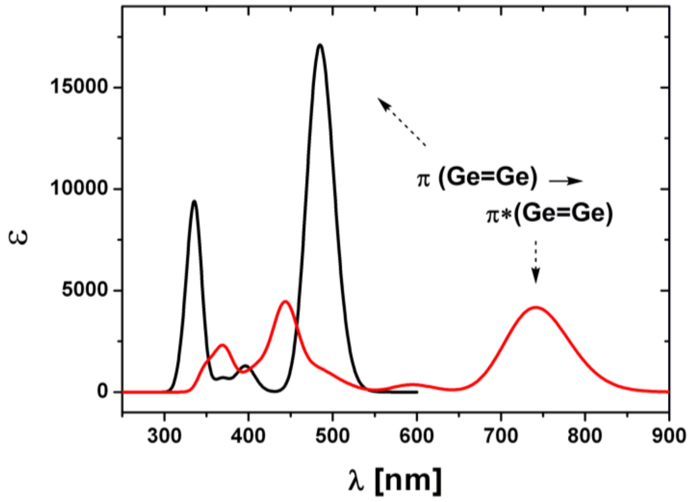

Figure S-1. Calculated gas phase UV spectra for digermene **2** (black trace, λ = 300-600 nm, first 10 excited states were collected) and its radical anion [**2**]•- (red trace, λ = 340-900 nm, first 20 excited states were collected). (At TD/B3LYP/def2tzvp//M062X/6-311+G(d,p)(Si,C,H);def2tzvp(Ge); individual excitations broadened with Gaussians with a band-width of 0.1 eV).

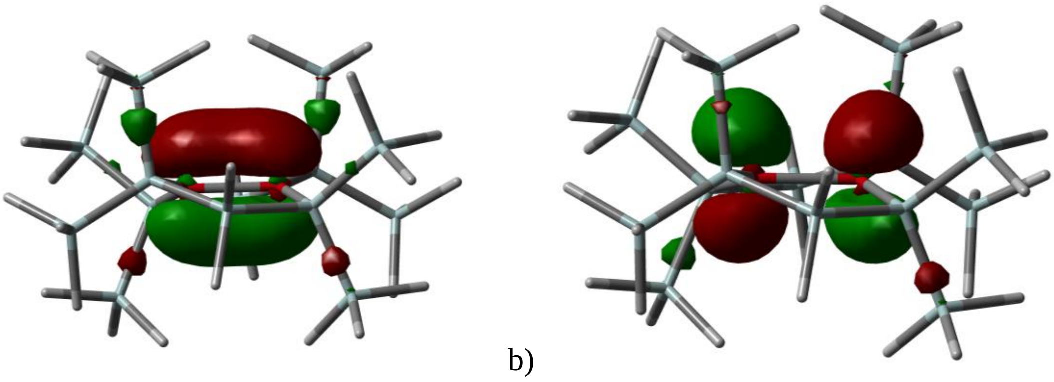

a) b)

Figure S-2. Calculated surface diagrams for a) HOMO of **2** (E(HOMO) = -4.6 eV) b) LUMO of **2** (E(LUMO) = -1.7 eV.(Color code: red (Ge), turquoise (Si), gray (C), hydrogen atoms are not shown. Surface isodensity value: 0.04, calculated at B3LYP/def2tzvp//M062X/ 6-311+G(d,p) (Si,C,H);def2tzvp(Ge)).

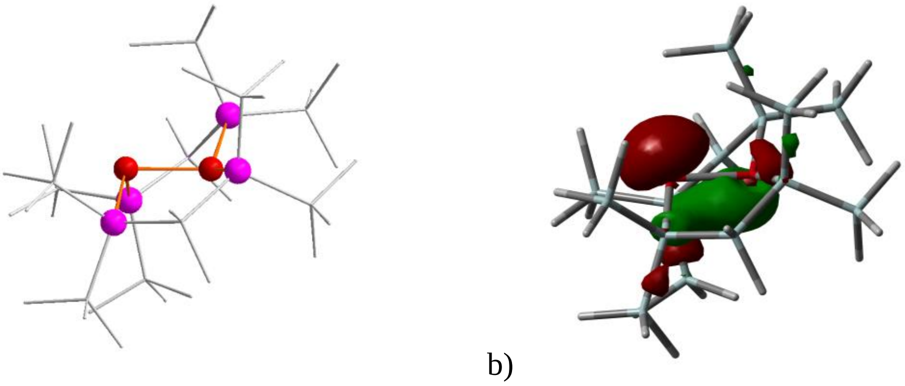

a) b)

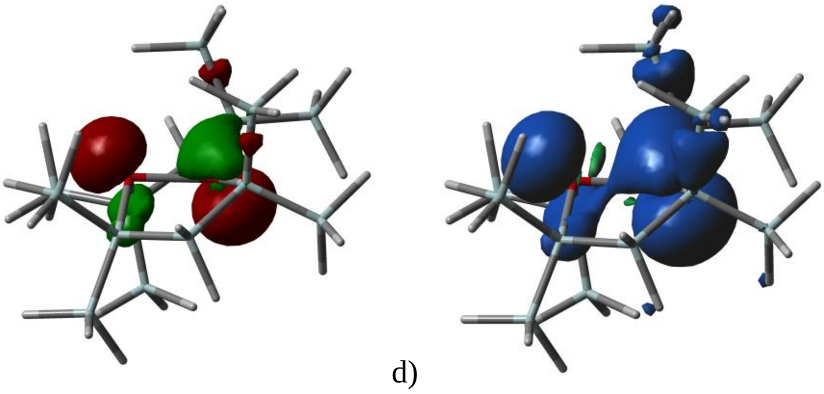

c) d)

Figure S-3. a) Ball and stick representation of the calculated molecular structures of digermene radical anion $[\mathbf{2}]^{\bullet-}$. the Si_2Ge-$GeSi_2$ core is accentuated, the polysilane backbone is shown in the wireframe mode. (See Table S-3 for selected structural parameter; color code: red (Ge), violet (Si). Calculated at M062X/6-311+G(d,p) (Si,C,H); def2tzvp(Ge)). b) Calculated surface diagrams for radical anion $[\mathbf{2}]^{\bullet-}$. b) SOMO-1 (surface isodensity value: 0.04) c) SOMO (surface isodensity value: 0.04). d) Calculated SCF spin density (difference between α and β spins) of radical anion $[\mathbf{2}]^{\bullet-}$. (blue positive spin density, green negative spin

density, surface isodensity value: 0.0008, color code: red (Ge), turquoise (Si) gray (C), hydrogen atoms are not shown. Calculated at B3LYP/def2tzvp//M062X/6-311+G(d,p)(Si,C,H);def2tzvp(Ge)).

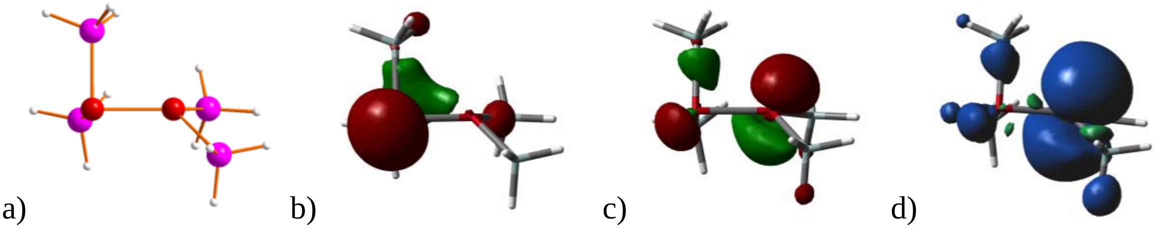

a) b) c) d)

Figure S-4. a) Ball and stick representation of the calculated molecular structures of digermene radical anion [**25**]$^{\bullet-}$. (See Table S2 for selected structural parameter; color code: red (Ge), violet (Si). Calculated at /M062X/6-311+G(d,p) (Si,C,H);def2tzvp(Ge)). b) Calculated surface diagrams for radical anion [**25**]$^{\bullet-}$. b) SOMO-1 (surface isodensity value: 0.05) c) SOMO (surface isodensity value: 0.05). d) Calculated SCF spin density (difference between α and β spins) of radical anion [**25**]$^{\bullet-}$. (blue positive spin density, green negative spin density, surface isodensity value: 0.0008, color code: red (Ge), turquoise (Si) gray (C), white (H). Calculated at B3LYP/def2tzvp//M062X/ 6-311+G(d,p) (Si,C,H);def2tzvp(Ge)).

Table S-3. Selected structural data of digermenes **2**, **11** and **14**, of radical anion [**2**]$^{\bullet-}$ and its radical cation [**2**]$^{\bullet+}$. Experimental data from XRD measurements are given in bold.

	**2(XRD)**	**2**	[**2**]$^{\bullet-}$	[**2**]$^{\bullet+}$	**11**	**14**	[**25**]$^{\bullet-}$
d(Ge=Ge) [pm]	**226.6**	228.4	246.6	239.0	231.5	233.8	250.4
α(SiGeSi) [°]	**139.98**	141.4; 141.4	118.6; 129.3	143.7; 143.7	90.8	105.3	90.2, 104.6
β(Si-Ge/SiGeSi) [°][a]	**2.5**; **2.5**	5.8; 5.8	92.5; 50.1	1.9; 1.9	35.6; 35.6	11.3; 11.3	88.9; 39.4
τ(SiGeSi/SiGeSi) [°][b]	**16.2**	18.8	12.5	18.8	0.0	0.0	73.9

[a] *trans*-bent angle β as defined in Figure S-5. [b] twist angle τ as defined in Figure S-5.

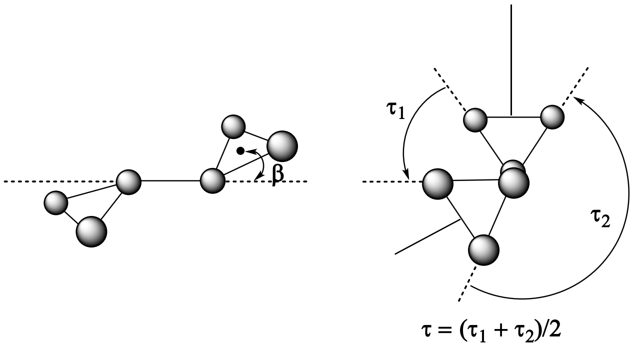

Figure S-5. Definition of the *trans*-bent angle β and of the twist angle τ.

Table S-4. Calculated molecular structures at M06-2X/def2-tzvp(Ge), 6-311+G(d,p) (P,Si,C,H) in the form of their Cartesian coordinates.

Compound 2

Standard orientation:

Center Number	Atomic Number	Atomic Type	Coordinates (Angstroms) X	Y	Z
1	32	0	1.141878	0.002163	0.056342
2	32	0	-1.141877	-0.002162	0.056344
3	14	0	1.880689	2.251678	0.348173
4	14	0	1.973478	-2.191271	-0.387232
5	14	0	-1.880688	-2.251676	0.348174
6	14	0	-1.973478	2.191272	-0.387231
7	14	0	0.010579	3.493043	-0.391081
8	14	0	2.149992	2.492962	2.670768
9	14	0	3.809275	2.926637	-0.799306
10	14	0	-0.010579	-3.493041	-0.391081
11	14	0	3.678084	-2.960676	1.032889
12	14	0	2.879604	-1.983212	-2.555718
13	14	0	-2.149992	-2.492958	2.670770
14	14	0	-3.809275	-2.926641	-0.799300
15	14	0	-3.678085	2.960676	1.032889
16	14	0	-2.879603	1.983209	-2.555717
17	6	0	0.322035	4.063484	-2.172302
18	6	0	-0.154124	5.060946	0.664582
19	6	0	0.480166	2.343222	3.530958
20	6	0	2.923536	4.165878	3.085993
21	6	0	3.278396	1.131186	3.327263
22	6	0	3.975864	4.805044	-0.667122
23	6	0	5.323425	2.153249	0.019120
24	6	0	3.733445	2.449726	-2.621277
25	6	0	-0.322037	-4.063479	-2.172303
26	6	0	0.154124	-5.060946	0.664579
27	6	0	3.040378	-3.421980	2.744607
28	6	0	5.008987	-1.639411	1.234461
29	6	0	4.461309	-4.488828	0.242874
30	6	0	4.598252	-1.210957	-2.384092
31	6	0	3.094270	-3.645088	-3.431576
32	6	0	1.789560	-0.877371	-3.628725
33	6	0	-0.480165	-2.343232	3.530960
34	6	0	-2.923553	-4.165866	3.085994
35	6	0	-3.278385	-1.131173	3.327265
36	6	0	-3.975856	-4.805048	-0.667113
37	6	0	-5.323425	-2.153259	0.019131
38	6	0	-3.733454	-2.449733	-2.621272
39	6	0	-3.040381	3.421981	2.744608
40	6	0	-5.008988	1.639411	1.234460
41	6	0	-4.461310	4.488829	0.242874
42	6	0	-4.598252	1.210958	-2.384092
43	6	0	-3.094265	3.645084	-3.431578
44	6	0	-1.789559	0.877364	-3.628720
45	1	0	-0.483103	4.723625	-2.504961
46	1	0	0.373581	3.210419	-2.854072
47	1	0	1.261797	4.616951	-2.247086
48	1	0	0.723669	5.696976	0.510230
49	1	0	-0.216652	4.829406	1.729615
50	1	0	-1.040591	5.638435	0.387064
51	1	0	0.612645	2.357999	4.617296
52	1	0	-0.013955	1.406315	3.258296
53	1	0	-0.188839	3.164112	3.264249
54	1	0	2.356979	4.993091	2.650602
55	1	0	3.949875	4.225446	2.713284
56	1	0	2.954637	4.312237	4.170015
57	1	0	3.376302	1.209231	4.414541
58	1	0	4.279644	1.197562	2.893386
59	1	0	2.870865	0.142227	3.096031
60	1	0	4.901168	5.135684	-1.149416
61	1	0	4.007938	5.128721	0.376357
62	1	0	3.145069	5.323043	-1.152463
63	1	0	5.241455	1.066091	0.089186
64	1	0	5.447466	2.543511	1.033821
65	1	0	6.231477	2.390008	-0.543816
66	1	0	2.917505	2.971479	-3.127274
67	1	0	3.575374	1.377484	-2.751763
68	1	0	4.668564	2.716451	-3.123596

69	1	0	0.483101	-4.723618	-2.504965
70	1	0	-0.373584	-3.210412	-2.854071
71	1	0	-1.261798	-4.616945	-2.247088
72	1	0	-0.723668	-5.696977	0.510223
73	1	0	0.216649	-4.829409	1.729612
74	1	0	1.040593	-5.638433	0.387061
75	1	0	2.339761	-4.259236	2.698696
76	1	0	2.536179	-2.581724	3.228121
77	1	0	3.880312	-3.722295	3.379004
78	1	0	4.575839	-0.697274	1.577303
79	1	0	5.537895	-1.450934	0.297965
80	1	0	5.743993	-1.961264	1.979056
81	1	0	5.228979	-4.906720	0.901417
82	1	0	4.937523	-4.241741	-0.710088
83	1	0	3.718869	-5.269237	0.055673
84	1	0	5.310900	-1.950308	-2.007074
85	1	0	4.599476	-0.362321	-1.695651
86	1	0	4.960965	-0.860262	-3.355443
87	1	0	-0.612643	-2.358007	4.617297
88	1	0	0.013964	-1.406329	3.258297
89	1	0	0.188834	-3.164127	3.264250
90	1	0	-2.357007	-4.993086	2.650600
91	1	0	-3.949893	-4.225422	2.713287
92	1	0	-2.954654	-4.312226	4.170016
93	1	0	-3.376286	-1.209213	4.414544
94	1	0	-4.279635	-1.197545	2.893394
95	1	0	-2.870850	-0.142217	3.096027
96	1	0	-4.901164	-5.135691	-1.149399
97	1	0	-4.007920	-5.128724	0.376366
98	1	0	-3.145064	-5.323045	-1.152461
99	1	0	-5.241461	-1.066100	0.089194
100	1	0	-5.447458	-2.543519	1.033833
101	1	0	-6.231478	-2.390023	-0.543800
102	1	0	-2.917512	-2.971481	-3.127271
103	1	0	-3.575392	-1.377490	-2.751761
104	1	0	-4.668573	-2.716466	-3.123587
105	1	0	-2.339764	4.259237	2.698697
106	1	0	-2.536182	2.581724	3.228122
107	1	0	-3.880316	3.722295	3.379003
108	1	0	-4.575838	0.697273	1.577300
109	1	0	-5.537896	1.450935	0.297964
110	1	0	-5.743993	1.961262	1.979056
111	1	0	-5.228982	4.906719	0.901415
112	1	0	-4.937522	4.241742	-0.710089
113	1	0	-3.718871	5.269239	0.055675
114	1	0	-5.310899	1.950311	-2.007076
115	1	0	-4.599480	0.362323	-1.695649
116	1	0	-4.960965	0.860262	-3.355442
117	1	0	3.820927	-3.541977	-4.243542
118	1	0	2.154245	-3.988175	-3.868071
119	1	0	3.458145	-4.420296	-2.752509
120	1	0	2.251521	-0.727568	-4.609928
121	1	0	1.622801	0.101420	-3.171550
122	1	0	0.806449	-1.330319	-3.783573
123	1	0	-0.806446	1.330310	-3.783566
124	1	0	-1.622804	-0.101427	-3.171544
125	1	0	-2.251518	0.727562	-4.609924
126	1	0	-2.154240	3.988169	-3.868072
127	1	0	-3.820922	3.541972	-4.243545
128	1	0	-3.458140	4.420294	-2.752513

Compound [2]$^{+\cdot}$

Standard orientation:

Center Number	Atomic Number	Atomic Type	Coordinates (Angstroms) X	Y	Z
1	32	0	-1.191865	-0.089306	-0.019497
2	32	0	1.191886	0.089315	-0.019517
3	14	0	-1.699444	-2.390259	0.329376
4	14	0	-2.162028	2.063431	-0.407862
5	14	0	1.699451	2.390303	0.329168
6	14	0	2.162044	-2.063459	-0.407713
7	14	0	0.262335	-3.491409	-0.416790
8	14	0	-1.740813	-2.442655	2.694358
9	14	0	-3.623762	-3.298974	-0.677398

10	14	0	-0.262325	3.491383	-0.417107
11	14	0	-3.974249	2.627046	1.005693
12	14	0	-3.011019	1.680241	-2.590969
13	14	0	1.740784	2.442881	2.694146
14	14	0	3.623775	3.298956	-0.677651
15	14	0	3.974299	-2.627009	1.005827
16	14	0	3.010968	-1.680466	-2.590885
17	6	0	-0.019276	-4.092840	-2.187465
18	6	0	0.542871	-5.010645	0.674920
19	6	0	0.011087	-2.117270	3.309590
20	6	0	-2.320532	-4.144587	3.246741
21	6	0	-2.890427	-1.110963	3.367124
22	6	0	-3.506776	-5.157545	-0.391483
23	6	0	-5.166846	-2.631377	0.162095
24	6	0	-3.655784	-2.929793	-2.521691
25	6	0	0.019268	4.092632	-2.187846
26	6	0	-0.542868	5.010733	0.674443
27	6	0	-3.447717	3.189854	2.719574
28	6	0	-5.102952	1.131090	1.169338
29	6	0	-4.868449	4.035502	0.131800
30	6	0	-4.675927	0.814620	-2.411111
31	6	0	-3.255772	3.293716	-3.527649
32	6	0	-1.832949	0.563017	-3.552871
33	6	0	-0.011084	2.117346	3.309395
34	6	0	2.320309	4.144916	3.246415
35	6	0	2.890554	1.111377	3.367021
36	6	0	3.506723	5.157554	-0.391937
37	6	0	5.166854	2.631492	0.161954
38	6	0	3.655842	2.929604	-2.521910
39	6	0	3.447806	-3.189820	2.719718
40	6	0	5.102976	-1.131031	1.169454
41	6	0	4.868520	-4.035446	0.131924
42	6	0	4.675944	-0.814948	-2.411169
43	6	0	3.255575	-3.294014	-3.527475
44	6	0	1.832934	-0.563219	-3.552807
45	1	0	0.819198	-4.712391	-2.515196
46	1	0	-0.126244	-3.259376	-2.887634
47	1	0	-0.924127	-4.703416	-2.246172
48	1	0	-0.313320	-5.687157	0.592602
49	1	0	0.664225	-4.749779	1.727979
50	1	0	1.432634	-5.560809	0.356407
51	1	0	0.095505	-2.352111	4.374844
52	1	0	0.298961	-1.070847	3.177374
53	1	0	0.738632	-2.729956	2.773825
54	1	0	-1.690774	-4.938594	2.837749
55	1	0	-3.350237	-4.331537	2.931270
56	1	0	-2.288648	-4.217870	4.337665
57	1	0	-2.828382	-1.079892	4.459055
58	1	0	-3.932194	-1.304083	3.099250
59	1	0	-2.621581	-0.118207	2.990927
60	1	0	-4.398171	-5.653259	-0.786935
61	1	0	-3.439172	-5.393735	0.673349
62	1	0	-2.638180	-5.596214	-0.888682
63	1	0	-5.258862	-1.548429	0.057099
64	1	0	-5.171447	-2.867359	1.229720
65	1	0	-6.057811	-3.086013	-0.281010
66	1	0	-2.761519	-3.307260	-3.022330
67	1	0	-3.729471	-1.859024	-2.722923
68	1	0	-4.523717	-3.413318	-2.979858
69	1	0	-0.819217	4.712141	-2.515630
70	1	0	0.126238	3.259101	-2.887935
71	1	0	0.924110	4.703215	-2.246624
72	1	0	0.313310	5.687250	0.592051
73	1	0	-0.664215	4.749976	1.727531
74	1	0	-1.432643	5.560847	0.355875
75	1	0	-2.775202	4.050089	2.686010
76	1	0	-2.963476	2.391930	3.286490
77	1	0	-4.342615	3.492787	3.272183
78	1	0	-4.538332	0.237080	1.444452
79	1	0	-5.638779	0.925945	0.241100
80	1	0	-5.845186	1.310073	1.952932
81	1	0	-5.730483	4.358625	0.722676
82	1	0	-5.235087	3.728730	-0.850918
83	1	0	-4.216722	4.902349	-0.005110
84	1	0	-5.441900	1.505360	-2.049639
85	1	0	-4.629743	-0.030082	-1.719144
86	1	0	-5.005634	0.436515	-3.383759
87	1	0	-0.095534	2.352289	4.374624

88	1	0	-0.298837	1.070875	3.177288
89	1	0	-0.738701	2.729889	2.773564
90	1	0	1.690476	4.938828	2.837354
91	1	0	3.350001	4.331953	2.930954
92	1	0	2.288394	4.218273	4.337333
93	1	0	2.828498	1.080373	4.458953
94	1	0	3.932300	1.304624	3.099152
95	1	0	2.621852	0.118559	2.990888
96	1	0	4.398084	5.653263	-0.787471
97	1	0	3.439141	5.393857	0.672872
98	1	0	2.638092	5.596129	-0.889157
99	1	0	5.258873	1.548527	0.057130
100	1	0	5.171437	2.867640	1.229543
101	1	0	6.057825	3.086060	-0.281207
102	1	0	2.761487	3.306838	-3.022564
103	1	0	3.729750	1.858833	-2.723051
104	1	0	4.523662	3.413272	-2.980139
105	1	0	2.775345	-4.050099	2.686165
106	1	0	2.963517	-2.391919	3.286625
107	1	0	4.342723	-3.492691	3.272330
108	1	0	4.538351	-0.237050	1.444651
109	1	0	5.638732	-0.925829	0.241188
110	1	0	5.845271	-1.310031	1.952986
111	1	0	5.730574	-4.358541	0.722786
112	1	0	5.235132	-3.728666	-0.850802
113	1	0	4.216815	-4.902311	-0.004971
114	1	0	5.441884	-1.505708	-2.049667
115	1	0	4.629847	0.029820	-1.719279
116	1	0	5.005634	-0.436956	-3.383866
117	1	0	-3.934767	3.123884	-4.368625
118	1	0	-2.314550	3.669476	-3.932234
119	1	0	-3.694048	4.072603	-2.899366
120	1	0	-2.209539	0.419031	-4.570355
121	1	0	-1.735673	-0.426973	-3.094495
122	1	0	-0.831700	0.996384	-3.627256
123	1	0	0.831660	-0.996532	-3.627153
124	1	0	1.735726	0.426796	-3.094472
125	1	0	2.209510	-0.419298	-4.570307
126	1	0	2.314312	-3.669750	-3.931986
127	1	0	3.934535	-3.124273	-4.368498
128	1	0	3.693844	-4.072884	-2.899164

Compound [2]$^{-\cdot}$

Standard orientation:

Center Number	Atomic Number	Atomic Type	Coordinates (Angstroms) X	Y	Z
1	32	0	-0.929190	0.616473	0.952816
2	32	0	0.910328	-0.368342	-0.361995
3	14	0	-2.673614	-1.014147	0.299699
4	14	0	-0.701466	2.680683	-0.357838
5	14	0	2.741057	1.076997	0.229113
6	14	0	0.786453	-2.767821	-0.304898
7	14	0	-1.565631	-2.980985	-0.437856
8	14	0	-3.642631	-1.339328	2.429756
9	14	0	-4.601364	-0.896586	-1.060265
10	14	0	1.643323	3.041542	-0.508360
11	14	0	-1.776994	4.406022	0.802155
12	14	0	-1.624901	2.643818	-2.512214
13	14	0	3.244147	1.374417	2.502905
14	14	0	4.730232	0.829699	-0.976064
15	14	0	1.845842	-3.655407	1.579545
16	14	0	1.811006	-3.616908	-2.235003
17	6	0	-1.924197	-3.162988	-2.300935
18	6	0	-2.191149	-4.564655	0.409484
19	6	0	-2.402036	-2.050525	3.665166
20	6	0	-5.164482	-2.474061	2.538767
21	6	0	-4.179144	0.344821	3.109008
22	6	0	-5.512772	-2.565926	-1.050690
23	6	0	-5.819794	0.360138	-0.322758
24	6	0	-4.386136	-0.486161	-2.895481
25	6	0	2.097554	3.316409	-2.336715
26	6	0	2.260816	4.597710	0.402366
27	6	0	-0.958077	4.724012	2.477518
28	6	0	-3.567680	3.904649	1.163047

29	6	0	-1.811186	6.049281	-0.148393
30	6	0	-3.489950	2.956051	-2.351943
31	6	0	-1.034924	4.003417	-3.703412
32	6	0	-1.295491	0.979003	-3.343589
33	6	0	1.761496	2.078245	3.430882
34	6	0	4.687723	2.598360	2.659259
35	6	0	3.778933	-0.201140	3.399248
36	6	0	5.722591	2.446600	-1.032191
37	6	0	5.889272	-0.440207	-0.179102
38	6	0	4.373516	0.290631	-2.752804
39	6	0	1.046501	-2.976823	3.146410
40	6	0	3.664867	-3.136331	1.545146
41	6	0	1.779482	-5.548301	1.645453
42	6	0	3.694913	-3.565589	-2.027619
43	6	0	1.362593	-5.428971	-2.573201
44	6	0	1.377191	-2.599764	-3.769357
45	1	0	-1.396595	-4.023286	-2.722004
46	1	0	-1.591741	-2.266962	-2.833384
47	1	0	-2.991206	-3.293774	-2.495091
48	1	0	-3.276945	-4.651343	0.302774
49	1	0	-1.955822	-4.557309	1.477109
50	1	0	-1.728953	-5.451462	-0.035112
51	1	0	-2.821774	-2.020486	4.676071
52	1	0	-1.473947	-1.474910	3.660858
53	1	0	-2.163156	-3.090864	3.430525
54	1	0	-4.954352	-3.471966	2.144583
55	1	0	-6.028943	-2.070758	2.005734
56	1	0	-5.445099	-2.582681	3.591915
57	1	0	-4.656303	0.225172	4.087308
58	1	0	-4.884592	0.848451	2.443670
59	1	0	-3.307072	0.993370	3.231174
60	1	0	-6.490822	-2.444670	-1.529689
61	1	0	-5.676076	-2.940072	-0.039590
62	1	0	-4.966389	-3.332970	-1.604543
63	1	0	-5.341217	1.315617	-0.092362
64	1	0	-6.247013	-0.026004	0.607358
65	1	0	-6.643471	0.549761	-1.018680
66	1	0	-3.705520	-1.183342	-3.388982
67	1	0	-4.005689	0.521024	-3.057365
68	1	0	-5.364928	-0.564509	-3.381661
69	1	0	1.646668	4.241443	-2.705577
70	1	0	1.735430	2.489253	-2.954773
71	1	0	3.180481	3.390321	-2.467960
72	1	0	3.313618	4.772501	0.157222
73	1	0	2.181839	4.497306	1.486933
74	1	0	1.691696	5.482699	0.101089
75	1	0	0.062051	5.099059	2.364109
76	1	0	-0.917932	3.799090	3.059610
77	1	0	-1.530023	5.462820	3.048038
78	1	0	-3.596102	2.901354	1.598109
79	1	0	-4.178945	3.894712	0.257733
80	1	0	-4.025437	4.597420	1.876690
81	1	0	-2.306268	6.830341	0.437637
82	1	0	-2.350667	5.946172	-1.094065
83	1	0	-0.798185	6.390287	-0.379064
84	1	0	-3.655080	3.990312	-2.035037
85	1	0	-3.959787	2.301184	-1.614931
86	1	0	-3.997698	2.816631	-3.311896
87	1	0	2.044151	2.345956	4.454517
88	1	0	0.948232	1.348757	3.469203
89	1	0	1.363437	2.970237	2.941138
90	1	0	4.491448	3.526000	2.115506
91	1	0	5.610858	2.164248	2.263942
92	1	0	4.860445	2.849394	3.710875
93	1	0	3.971163	0.025637	4.453166
94	1	0	4.688562	-0.629171	2.970638
95	1	0	2.996990	-0.962417	3.353067
96	1	0	6.675336	2.285643	-1.547368
97	1	0	5.940554	2.804141	-0.022658
98	1	0	5.188256	3.241917	-1.556372
99	1	0	5.448787	-1.438195	-0.129020
100	1	0	6.148142	-0.140893	0.841210
101	1	0	6.818906	-0.508953	-0.753714
102	1	0	3.810660	1.061789	-3.285020
103	1	0	3.776227	-0.624614	-2.773270
104	1	0	5.303428	0.104979	-3.299581
105	1	0	0.093404	-3.473674	3.339888
106	1	0	0.842203	-1.906156	3.048659

107	1	0	1.692591	-3.130761	4.016617
108	1	0	3.749029	-2.079787	1.277332
109	1	0	4.231495	-3.717478	0.814419
110	1	0	4.128934	-3.272062	2.527189
111	1	0	2.241012	-5.920737	2.565599
112	1	0	2.313690	-5.989317	0.799368
113	1	0	0.747003	-5.906143	1.614146
114	1	0	4.037077	-4.379152	-1.382243
115	1	0	4.023165	-2.623369	-1.581578
116	1	0	4.190583	-3.673750	-2.997670
117	1	0	-1.755845	4.104020	-4.521714
118	1	0	-0.062742	3.768783	-4.139761
119	1	0	-0.957568	4.973792	-3.204591
120	1	0	-1.843005	0.880825	-4.286306
121	1	0	-1.584847	0.159405	-2.679630
122	1	0	-0.226485	0.866097	-3.547405
123	1	0	0.334507	-2.740947	-4.061589
124	1	0	1.528887	-1.532017	-3.588753
125	1	0	2.010552	-2.899586	-4.610502
126	1	0	0.295491	-5.557189	-2.768811
127	1	0	1.911177	-5.802342	-3.444100
128	1	0	1.620658	-6.057312	-1.716385

Compound 3

Standard orientation:

Center Number	Atomic Number	Atomic Type	Coordinates (Angstroms) X	Y	Z
1	14	0	-1.637685	-0.341207	0.186319
2	14	0	0.042842	-1.134503	1.642725
3	14	0	1.714260	-0.320701	0.191688
4	32	0	0.042460	0.188128	-1.584057
5	6	0	0.084637	-3.009142	1.921244
6	1	0	0.871823	-3.268753	2.635578
7	1	0	-0.869656	-3.362533	2.323276
8	1	0	0.282018	-3.546710	0.991318
9	6	0	0.043764	-0.323714	3.357609
10	1	0	-0.741134	-0.755542	3.984769
11	1	0	1.002977	-0.498361	3.854738
12	1	0	-0.114393	0.755721	3.300137
13	14	0	-2.939053	-1.997847	-0.846270
14	14	0	-3.199577	0.976797	1.347537
15	14	0	3.054259	-2.033024	-0.686236
16	14	0	3.179807	1.231558	1.164333
17	6	0	-1.840021	-3.392707	-1.484573
18	1	0	-1.052408	-3.001249	-2.134388
19	1	0	-1.367003	-3.941038	-0.665900
20	1	0	-2.432254	-4.104790	-2.068019
21	6	0	-4.763809	1.296974	0.331085
22	1	0	-4.559488	1.802818	-0.615882
23	1	0	-5.278288	0.360931	0.099254
24	1	0	-5.453574	1.923769	0.905507
25	6	0	2.022495	-3.151686	-1.804008
26	1	0	1.222092	-3.645773	-1.246643
27	1	0	1.560233	-2.581898	-2.614939
28	1	0	2.650439	-3.929685	-2.249564
29	6	0	2.363482	2.290901	2.505469
30	1	0	1.367512	2.644367	2.234421
31	1	0	2.264256	1.704002	3.422097
32	1	0	2.984700	3.162932	2.733891
33	6	0	4.437341	-1.280862	-1.734429
34	1	0	5.137586	-0.701091	-1.127223
35	1	0	5.005711	-2.064742	-2.244350
36	1	0	4.021048	-0.616557	-2.497293
37	6	0	-3.783820	-1.208824	-2.342035
38	1	0	-4.360059	-1.954996	-2.897840
39	1	0	-4.462834	-0.404417	-2.049841
40	1	0	-3.031681	-0.790385	-3.017198
41	6	0	-3.703214	-0.009031	2.880710
42	1	0	-2.906357	0.011541	3.627760
43	1	0	-4.600575	0.424616	3.333554
44	1	0	-3.913713	-1.053685	2.640884
45	6	0	3.960885	2.341101	-0.156123
46	1	0	4.681858	3.028420	0.297640
47	1	0	4.494006	1.730840	-0.890097

48	1	0	3.226762	2.937858	-0.701242
49	6	0	-2.589224	2.658003	1.972496
50	1	0	-2.490376	3.395116	1.171821
51	1	0	-3.316212	3.052217	2.689998
52	1	0	-1.629176	2.573980	2.489643
53	6	0	-4.257653	-2.755897	0.279573
54	1	0	-4.978713	-2.013519	0.631706
55	1	0	-4.812214	-3.529286	-0.261170
56	1	0	-3.802432	-3.224034	1.156702
57	6	0	3.811833	-3.108190	0.670126
58	1	0	3.026126	-3.603104	1.246834
59	1	0	4.445630	-3.884429	0.230128
60	1	0	4.422258	-2.525890	1.363456
61	6	0	4.600509	0.315609	2.013417
62	1	0	5.215057	-0.230852	1.293681
63	1	0	5.246656	1.025434	2.539578
64	1	0	4.220701	-0.402485	2.745348
65	15	0	-0.115700	2.570057	-1.539014
66	6	0	0.262017	3.673619	-0.126802
67	1	0	-0.004966	4.705076	-0.372573
68	1	0	-0.300174	3.349967	0.747900
69	1	0	1.326045	3.621661	0.102066
70	6	0	-1.823475	3.041638	-1.996929
71	1	0	-2.127143	2.466198	-2.873190
72	1	0	-2.490878	2.792589	-1.170786
73	1	0	-1.888934	4.110297	-2.213564
74	6	0	0.900061	3.277554	-2.889298
75	1	0	0.591292	2.823650	-3.831898
76	1	0	0.782389	4.362177	-2.943233
77	1	0	1.949436	3.031567	-2.718370

Compound 4

Standard orientation:

Center Number	Atomic Number	Atomic Type	Coordinates (Angstroms) X	Y	Z
1	14	0	-1.590220	-0.045611	0.097915
2	14	0	-0.000017	-0.000074	1.823568
3	14	0	1.590184	0.045588	0.097918
4	32	0	-0.000017	0.000022	-1.777629
5	6	0	0.028611	-1.525476	2.946603
6	1	0	0.921486	-1.528650	3.578470
7	1	0	-0.847485	-1.519162	3.602305
8	1	0	0.010416	-2.453779	2.370745
9	6	0	-0.028636	1.525228	2.946739
10	1	0	-0.921484	1.528328	3.578644
11	1	0	0.847487	1.518878	3.602404
12	1	0	-0.010487	2.453585	2.370966
13	14	0	-3.498639	-1.406638	0.055784
14	14	0	-2.255380	2.188127	-0.271571
15	14	0	2.255453	-2.188095	-0.271695
16	14	0	3.498571	1.406667	0.055855
17	6	0	-3.121103	-3.062292	0.877042
18	1	0	-2.363932	-3.615413	0.316017
19	1	0	-2.753562	-2.925814	1.897277
20	1	0	-4.022912	-3.680583	0.921833
21	6	0	-0.746606	3.241922	-0.720925
22	1	0	0.022362	3.187059	0.053857
23	1	0	-0.289656	2.921872	-1.664984
24	1	0	-1.034279	4.291391	-0.838956
25	6	0	0.746783	-3.241966	-0.721220
26	1	0	-0.022165	-3.187366	0.053600
27	1	0	0.289759	-2.921795	-1.665201
28	1	0	1.034593	-4.291372	-0.839470
29	6	0	3.120995	3.062343	0.877053
30	1	0	2.363935	3.615504	0.315919
31	1	0	2.753308	2.925899	1.897241
32	1	0	4.022832	3.680585	0.921960
33	6	0	3.468989	-2.240177	-1.716383
34	1	0	4.392174	-1.704794	-1.479068
35	1	0	3.734511	-3.275453	-1.952364
36	1	0	3.037918	-1.787590	-2.613232
37	6	0	-4.000705	-1.698593	-1.736589
38	1	0	-4.906714	-2.309769	-1.790470
39	1	0	-4.194800	-0.755942	-2.254098

40	1	0	-3.206377	-2.218322	-2.278788
41	6	0	-3.468834	2.240390	-1.716320
42	1	0	-4.392037	1.704990	-1.479109
43	1	0	-3.734334	3.275694	-1.952201
44	1	0	-3.037724	1.787898	-2.613198
45	6	0	4.000717	1.698593	-1.736501
46	1	0	4.906710	2.309795	-1.790355
47	1	0	4.194863	0.755936	-2.253980
48	1	0	3.206399	2.218287	-2.278750
49	6	0	-3.085786	2.939524	1.245380
50	1	0	-2.403763	2.982089	2.096994
51	1	0	-3.416596	3.959389	1.023709
52	1	0	-3.961670	2.357743	1.542528
53	6	0	-4.925080	-0.586617	0.983385
54	1	0	-5.228995	0.345920	0.500873
55	1	0	-5.795965	-1.248850	1.008663
56	1	0	-4.646945	-0.356245	2.015150
57	6	0	3.085805	-2.939582	1.245243
58	1	0	2.403661	-2.982439	2.096745
59	1	0	3.416857	-3.959339	1.023440
60	1	0	3.961524	-2.357675	1.542627
61	6	0	4.924976	0.586685	0.983545
62	1	0	5.228857	-0.345918	0.501140
63	1	0	5.795888	1.248886	1.008755
64	1	0	4.646827	0.356438	2.015335

TS 4/10

Standard orientation:

Center Number	Atomic Number	Atomic Type	Coordinates (Angstroms) X	Y	Z
1	32	0	0.240783	0.563347	-1.509962
2	14	0	-0.040220	-1.418218	1.452010
3	14	0	1.571958	-0.693273	-0.097526
4	14	0	-1.507784	-0.200236	0.071417
5	14	0	3.893571	-1.133960	0.096980
6	14	0	1.533203	2.392851	-0.441031
7	14	0	-2.787623	-1.720263	-1.172101
8	14	0	-2.924348	1.410622	1.010021
9	6	0	4.021763	-2.816874	0.941361
10	1	0	3.490275	-3.589136	0.379563
11	1	0	5.068330	-3.124594	1.027648
12	1	0	3.596626	-2.781567	1.947994
13	6	0	4.680390	-1.240923	-1.611104
14	1	0	4.207141	-2.028454	-2.202624
15	1	0	4.589583	-0.307854	-2.170816
16	1	0	5.744410	-1.479967	-1.521171
17	6	0	4.797791	0.133033	1.162974
18	1	0	4.404543	0.142014	2.182691
19	1	0	5.856499	-0.140100	1.216930
20	1	0	4.731004	1.146894	0.763107
21	6	0	-4.018077	-0.802582	-2.271821
22	1	0	-3.517054	-0.036850	-2.870301
23	1	0	-4.512589	-1.497059	-2.958001
24	1	0	-4.794707	-0.313563	-1.677047
25	6	0	-1.645945	-2.744771	-2.272528
26	1	0	-1.157503	-2.112612	-3.018696
27	1	0	-0.858463	-3.235611	-1.693688
28	1	0	-2.211722	-3.519758	-2.798491
29	6	0	-3.750237	-2.884952	-0.037060
30	1	0	-4.382980	-2.328588	0.658867
31	1	0	-4.395206	-3.542638	-0.628181
32	1	0	-3.078083	-3.514707	0.550980
33	6	0	-3.546213	2.546144	-0.362545
34	1	0	-4.121498	1.986431	-1.103874
35	1	0	-4.192929	3.327554	0.048121
36	1	0	-2.717970	3.030394	-0.885751
37	6	0	-4.411557	0.563945	1.810973
38	1	0	-5.007365	0.037387	1.060377
39	1	0	-4.100009	-0.164322	2.564418
40	1	0	-5.059348	1.299729	2.297261
41	6	0	-2.047068	2.458035	2.311915
42	1	0	-1.626259	1.840954	3.109822
43	1	0	-1.235807	3.040846	1.868668
44	1	0	-2.751266	3.162191	2.766048

45	6	0	1.859064	2.401888	1.423678
46	1	0	0.915852	2.294650	1.965551
47	1	0	2.520561	1.592705	1.741424
48	1	0	2.312102	3.353112	1.724626
49	6	0	3.159455	2.561223	-1.377670
50	1	0	2.963838	2.779580	-2.429894
51	1	0	3.753443	3.380414	-0.959509
52	1	0	3.759203	1.650494	-1.332720
53	6	0	0.468537	3.915534	-0.776693
54	1	0	0.957398	4.809393	-0.374228
55	1	0	0.310850	4.052968	-1.848356
56	1	0	-0.510616	3.825291	-0.300309
57	6	0	-0.307980	-3.280322	1.652135
58	1	0	0.571426	-3.745678	2.106280
59	1	0	-1.165076	-3.468328	2.305696
60	1	0	-0.497386	-3.769231	0.694440
61	6	0	0.262114	-0.697072	3.177653
62	1	0	1.095261	-1.212737	3.663980
63	1	0	0.493459	0.368581	3.144558
64	1	0	-0.627843	-0.834473	3.799033

Compound 10

Standard orientation:

Center Number	Atomic Number	Atomic Type	Coordinates (Angstroms) X	Y	Z
1	14	0	1.440575	-0.558471	0.002541
2	14	0	-0.111038	-2.344671	-0.071898
3	14	0	-1.872398	-0.814179	-0.065919
4	32	0	-0.467074	0.902845	-0.090493
5	6	0	-0.046175	-3.517042	1.414186
6	1	0	-0.912273	-4.185031	1.411690
7	1	0	0.854893	-4.136207	1.376012
8	1	0	-0.047142	-2.968021	2.357787
9	6	0	-0.003170	-3.414994	-1.630568
10	1	0	0.883524	-4.054614	-1.600688
11	1	0	-0.881249	-4.062816	-1.706031
12	1	0	0.041361	-2.800841	-2.532675
13	14	0	2.502949	-0.356103	2.080873
14	14	0	2.820608	-0.415420	-1.888546
15	14	0	-4.210091	-0.896623	-0.019896
16	6	0	1.188575	-0.058434	3.400643
17	1	0	0.702663	0.909220	3.246410
18	1	0	0.406177	-0.820885	3.372593
19	1	0	1.636447	-0.060923	4.399020
20	6	0	1.764649	0.118906	-3.356244
21	1	0	0.908024	-0.546835	-3.490454
22	1	0	1.373872	1.128914	-3.204357
23	1	0	2.351050	0.115065	-4.279998
24	6	0	-4.847179	0.866259	0.152615
25	1	0	-4.481364	1.321310	1.076306
26	1	0	-4.517890	1.485739	-0.685023
27	1	0	-5.940886	0.879720	0.176384
28	6	0	-4.874559	-1.670686	-1.604018
29	1	0	-4.491512	-2.685375	-1.736608
30	1	0	-5.967105	-1.723541	-1.572897
31	1	0	-4.586074	-1.085673	-2.480035
32	6	0	3.687398	1.115399	2.084722
33	1	0	4.085040	1.279846	3.090997
34	1	0	4.532232	0.956458	1.411134
35	1	0	3.179430	2.031639	1.771221
36	6	0	4.206553	0.842093	-1.633565
37	1	0	4.940566	0.472234	-0.913169
38	1	0	4.728257	1.027470	-2.577457
39	1	0	3.826007	1.797722	-1.264089
40	6	0	3.600111	-2.095134	-2.251870
41	1	0	2.834840	-2.840527	-2.479644
42	1	0	4.275999	-2.031458	-3.109944
43	1	0	4.176178	-2.454824	-1.395133
44	6	0	3.468443	-1.925394	2.490928
45	1	0	4.239042	-2.121176	1.740399
46	1	0	3.961240	-1.833791	3.463585
47	1	0	2.805385	-2.793443	2.527938
48	6	0	-4.767736	-1.932880	1.452619
49	1	0	-4.402563	-1.511578	2.391846

50	1	0	-5.860167	-1.975512	1.498773
51	1	0	-4.393911	-2.956951	1.376104
52	14	0	-0.571193	3.295113	-0.065625
53	6	0	-0.072196	3.894487	1.649018
54	1	0	0.931540	3.552968	1.913013
55	1	0	-0.077453	4.988210	1.685735
56	1	0	-0.765288	3.525156	2.408555
57	6	0	0.652805	3.938458	-1.344728
58	1	0	0.705253	5.030894	-1.312344
59	1	0	1.655715	3.545615	-1.158300
60	1	0	0.357239	3.643214	-2.354268
61	6	0	-2.309327	3.887323	-0.471109
62	1	0	-2.622007	3.543181	-1.459562
63	1	0	-3.032194	3.518216	0.260012
64	1	0	-2.347444	4.980950	-0.464219

Compound 11

Standard orientation:

Center Number	Atomic Number	Atomic Type	Coordinates (Angstroms) X	Y	Z
1	14	0	-1.483794	-0.076519	2.846133
2	14	0	0.305601	0.772089	4.149834
3	14	0	1.966513	0.109834	2.598887
4	14	0	-1.966513	-0.109834	-2.598887
5	14	0	-0.305601	-0.772089	-4.149834
6	14	0	1.483794	0.076519	-2.846133
7	32	0	-0.142875	0.413150	-1.071968
8	32	0	0.142875	-0.413150	1.071968
9	14	0	2.848405	-1.914118	3.387311
10	14	0	-2.686170	-1.941132	3.671995
11	14	0	-3.000647	1.631181	2.282512
12	14	0	3.565796	1.721508	1.968898
13	14	0	3.000647	-1.631181	-2.282512
14	14	0	2.686170	1.941132	-3.671995
15	14	0	-3.565796	-1.721508	-1.968898
16	14	0	-2.848405	1.914118	-3.387311
17	6	0	-3.058755	-2.431556	-0.299310
18	1	0	-2.072332	-2.898149	-0.383507
19	1	0	-2.979006	-1.644041	0.455874
20	1	0	-3.769489	-3.185191	0.054537
21	6	0	-5.304247	-0.990492	-1.891434
22	1	0	-5.611365	-0.614316	-2.871287
23	1	0	-6.015885	-1.769280	-1.598569
24	1	0	-5.383816	-0.174536	-1.172066
25	6	0	-3.612334	-3.118975	-3.237499
26	1	0	-4.445325	-3.792410	-3.012381
27	1	0	-3.756864	-2.734346	-4.250940
28	1	0	-2.691831	-3.705486	-3.226343
29	6	0	-3.802062	1.600619	-4.986101
30	1	0	-4.637085	0.915576	-4.818145
31	1	0	-4.210821	2.537186	-5.377975
32	1	0	-3.158249	1.164861	-5.753607
33	6	0	-3.989325	2.756027	-2.146637
34	1	0	-4.364359	3.695974	-2.563707
35	1	0	-4.847573	2.127309	-1.897845
36	1	0	-3.456200	2.983935	-1.219869
37	6	0	-1.392801	3.066261	-3.720074
38	1	0	-0.881368	3.297883	-2.781188
39	1	0	-0.657651	2.613152	-4.391939
40	1	0	-1.725149	4.008795	-4.166172
41	6	0	-0.195915	-2.660884	-4.257946
42	1	0	-1.006298	-3.047180	-4.882160
43	1	0	0.754167	-2.979932	-4.696169
44	1	0	-0.284855	-3.110756	-3.266677
45	6	0	-0.504402	-0.125307	-5.921454
46	1	0	-1.363478	-0.610424	-6.394743
47	1	0	-0.661907	0.954185	-5.961416
48	1	0	0.384824	-0.359924	-6.513539
49	6	0	2.175611	-3.196996	-1.631858
50	1	0	1.430984	-2.969597	-0.863679
51	1	0	1.690203	-3.761874	-2.429240
52	1	0	2.940708	-3.841098	-1.185854
53	6	0	4.014417	-2.106762	-3.807905
54	1	0	4.740677	-2.885513	-3.555154

55	1	0	3.361900	-2.501640	-4.591546
56	1	0	4.561689	-1.259799	-4.227381
57	6	0	4.132072	-0.999302	-0.913042
58	1	0	4.871852	-1.761362	-0.646773
59	1	0	4.663281	-0.089155	-1.197390
60	1	0	3.538309	-0.785915	-0.017068
61	6	0	2.569578	1.892495	-5.555795
62	1	0	3.172227	2.691588	-5.998301
63	1	0	2.932027	0.938072	-5.948334
64	1	0	1.537625	2.020619	-5.890988
65	6	0	4.517130	1.829277	-3.211524
66	1	0	4.958457	0.861409	-3.457542
67	1	0	5.061249	2.597945	-3.770264
68	1	0	4.677182	2.018380	-2.147753
69	6	0	2.064930	3.611039	-3.050615
70	1	0	1.205189	3.959643	-3.624359
71	1	0	1.780055	3.570085	-1.996845
72	1	0	2.864232	4.351455	-3.155276
73	6	0	3.058755	2.431556	0.299310
74	1	0	2.979006	1.644041	-0.455874
75	1	0	2.072332	2.898149	0.383507
76	1	0	3.769489	3.185191	-0.054537
77	6	0	5.304247	0.990492	1.891434
78	1	0	6.015885	1.769280	1.598569
79	1	0	5.611365	0.614316	2.871287
80	1	0	5.383816	0.174536	1.172066
81	6	0	3.612334	3.118975	3.237499
82	1	0	3.756864	2.734346	4.250940
83	1	0	4.445325	3.792410	3.012381
84	1	0	2.691831	3.705486	3.226343
85	6	0	0.195915	2.660884	4.257946
86	1	0	-0.754167	2.979932	4.696169
87	1	0	1.006298	3.047180	4.882160
88	1	0	0.284855	3.110756	3.266677
89	6	0	0.504402	0.125307	5.921454
90	1	0	-0.384824	0.359924	6.513539
91	1	0	0.661907	-0.954185	5.961416
92	1	0	1.363478	0.610424	6.394743
93	6	0	-4.014417	2.106762	3.807905
94	1	0	-3.361900	2.501640	4.591546
95	1	0	-4.740677	2.885513	3.555154
96	1	0	-4.561689	1.259799	4.227381
97	6	0	-2.175611	3.196996	1.631858
98	1	0	-1.690203	3.761874	2.429240
99	1	0	-1.430984	2.969597	0.863679
100	1	0	-2.940708	3.841098	1.185854
101	6	0	-4.132072	0.999302	0.913042
102	1	0	-4.663281	0.089155	1.197390
103	1	0	-4.871852	1.761362	0.646773
104	1	0	-3.538309	0.785915	0.017068
105	6	0	-4.517130	-1.829277	3.211524
106	1	0	-5.061249	-2.597945	3.770264
107	1	0	-4.958457	-0.861409	3.457542
108	1	0	-4.677182	-2.018380	2.147753
109	6	0	-2.569578	-1.892495	5.555795
110	1	0	-2.932027	-0.938072	5.948334
111	1	0	-3.172227	-2.691588	5.998301
112	1	0	-1.537625	-2.020619	5.890988
113	6	0	-2.064930	-3.611039	3.050615
114	1	0	-1.780055	-3.570085	1.996845
115	1	0	-1.205189	-3.959643	3.624359
116	1	0	-2.864232	-4.351455	3.155276
117	6	0	3.802062	-1.600619	4.986101
118	1	0	4.210821	-2.537186	5.377975
119	1	0	4.637085	-0.915576	4.818145
120	1	0	3.158249	-1.164861	5.753607
121	6	0	3.989325	-2.756027	2.146637
122	1	0	4.847573	-2.127309	1.897845
123	1	0	4.364359	-3.695974	2.563707
124	1	0	3.456200	-2.983935	1.219869
125	6	0	1.392801	-3.066261	3.720074
126	1	0	0.657651	-2.613152	4.391939
127	1	0	0.881368	-3.297883	2.781188
128	1	0	1.725149	-4.008795	4.166172

Compound 12

Standard orientation:

Center Number	Atomic Number	Atomic Type	Coordinates (Angstroms) X	Y	Z
1	32	0	-0.609413	0.044641	-0.002552
2	14	0	-3.673390	1.356542	-0.764550
3	14	0	-2.484112	-0.553307	-1.493111
4	14	0	-1.909420	2.032214	0.654185
5	32	0	1.142673	0.903823	-1.596027
6	14	0	0.453062	-1.546517	1.529754
7	14	0	3.163300	0.299142	-0.345325
8	14	0	2.531987	-0.452515	1.792538
9	14	0	-3.649566	-2.524898	-0.965089
10	14	0	-2.111019	-0.503291	-3.820145
11	14	0	-2.547381	2.056376	2.910278
12	14	0	-0.901654	4.071582	0.072678
13	14	0	-0.153257	-2.315743	3.681880
14	14	0	0.831344	-3.474731	0.243223
15	14	0	4.541460	2.247096	-0.252044
16	14	0	4.500588	-1.058359	-1.770728
17	6	0	3.893780	-1.386781	2.727597
18	1	0	4.828550	-0.822838	2.652168
19	1	0	3.640788	-1.469121	3.788532
20	1	0	4.071354	-2.389496	2.335040
21	6	0	2.136296	1.102112	2.804769
22	1	0	3.034331	1.676556	3.037221
23	1	0	1.446089	1.748211	2.254437
24	1	0	1.659609	0.823600	3.747421
25	6	0	0.606913	-2.990500	-1.564868
26	1	0	-0.424863	-2.687973	-1.752939
27	1	0	1.243148	-2.133831	-1.807131
28	1	0	0.859126	-3.800036	-2.256916
29	6	0	2.593654	-4.087849	0.540307
30	1	0	2.723634	-4.382782	1.585223
31	1	0	2.825152	-4.953254	-0.088698
32	1	0	3.323238	-3.303199	0.321861
33	6	0	-0.346565	-4.897608	0.646889
34	1	0	-1.395494	-4.601150	0.578657
35	1	0	-0.185477	-5.720280	-0.057526
36	1	0	-0.172128	-5.284683	1.654003
37	6	0	-0.026647	-0.976214	5.007572
38	1	0	-0.535162	-0.051661	4.727703
39	1	0	-0.474333	-1.340707	5.937641
40	1	0	1.020865	-0.741982	5.215280
41	6	0	1.159092	-3.623898	4.081052
42	1	0	2.175460	-3.261805	3.911651
43	1	0	1.075450	-3.912347	5.134088
44	1	0	1.020062	-4.527078	3.481117
45	6	0	-1.812797	-3.197524	3.854260
46	1	0	-2.673607	-2.542896	3.713131
47	1	0	-1.897976	-4.027125	3.148597
48	1	0	-1.863752	-3.612490	4.866717
49	6	0	-3.529742	-2.778744	0.898981
50	1	0	-2.494869	-2.695710	1.241198
51	1	0	-4.101031	-2.004207	1.420467
52	1	0	-3.916972	-3.752655	1.214526
53	6	0	-5.470780	-2.361545	-1.441671
54	1	0	-5.965271	-3.334195	-1.354270
55	1	0	-5.989807	-1.662157	-0.783690
56	1	0	-5.591647	-2.015015	-2.470597
57	6	0	-2.985585	-4.033215	-1.892882
58	1	0	-3.579934	-4.913313	-1.625757
59	1	0	-3.078611	-3.886846	-2.972981
60	1	0	-1.940886	-4.258293	-1.674073
61	6	0	-0.757960	-1.685947	-4.397434
62	1	0	-0.953751	-2.717769	-4.096809
63	1	0	-0.705370	-1.652430	-5.490561
64	1	0	0.222446	-1.400973	-4.006375
65	6	0	-3.737177	-1.039496	-4.625871
66	1	0	-3.660303	-0.959028	-5.714918
67	1	0	-3.991686	-2.074836	-4.384287
68	1	0	-4.566223	-0.403849	-4.301682
69	6	0	-1.695776	1.213686	-4.482213
70	1	0	-2.571193	1.866016	-4.472519
71	1	0	-0.894654	1.690934	-3.913778
72	1	0	-1.359924	1.122679	-5.520334

73	6	0	-5.278358	0.963309	0.163377
74	1	0	-6.043541	0.628723	-0.542647
75	1	0	-5.141730	0.181293	0.913607
76	1	0	-5.657354	1.858592	0.663385
77	6	0	-4.121795	2.617953	-2.100815
78	1	0	-4.847128	2.194484	-2.802342
79	1	0	-4.572583	3.504480	-1.643710
80	1	0	-3.244517	2.934452	-2.665096
81	6	0	-3.050937	0.304242	3.399832
82	1	0	-4.050215	0.078438	3.017397
83	1	0	-2.359221	-0.427044	2.974073
84	1	0	-3.067925	0.170787	4.485831
85	6	0	-1.138841	2.677651	4.001449
86	1	0	-0.929022	3.729841	3.787259
87	1	0	-1.412434	2.601475	5.058762
88	1	0	-0.214662	2.118005	3.848912
89	6	0	-4.019430	3.208815	3.175969
90	1	0	-4.302250	3.210313	4.233676
91	1	0	-3.782479	4.236896	2.891891
92	1	0	-4.889229	2.888192	2.598708
93	6	0	-1.782827	5.488758	0.959261
94	1	0	-1.350350	6.449502	0.663571
95	1	0	-2.846891	5.508398	0.708182
96	1	0	-1.693592	5.399231	2.045147
97	6	0	0.899776	4.048978	0.634912
98	1	0	1.411128	4.973472	0.347585
99	1	0	0.969514	3.946049	1.721863
100	1	0	1.457040	3.215311	0.191828
101	6	0	-0.945808	4.421530	-1.780183
102	1	0	-0.652086	3.554010	-2.376901
103	1	0	-1.945417	4.729605	-2.094621
104	1	0	-0.255419	5.238239	-2.014181
105	6	0	4.131481	3.340621	-1.737401
106	1	0	4.146114	2.786619	-2.678223
107	1	0	3.133128	3.774953	-1.627598
108	1	0	4.849773	4.163314	-1.811485
109	6	0	4.349328	3.292103	1.310663
110	1	0	4.822131	2.801011	2.165099
111	1	0	4.875507	4.238338	1.143710
112	1	0	3.317345	3.521821	1.571280
113	6	0	6.387279	1.822066	-0.249383
114	1	0	6.726238	1.236570	-1.103742
115	1	0	6.955416	2.758208	-0.235354
116	1	0	6.641853	1.271428	0.660756
117	6	0	5.947321	-1.732702	-0.761244
118	1	0	5.584349	-2.445010	-0.014642
119	1	0	6.646708	-2.262972	-1.415397
120	1	0	6.496456	-0.950525	-0.235960
121	6	0	3.726665	-2.543838	-2.643786
122	1	0	2.931731	-2.252461	-3.334426
123	1	0	4.523978	-3.006569	-3.236106
124	1	0	3.333761	-3.301730	-1.966449
125	6	0	5.086951	0.030446	-3.202120
126	1	0	5.633552	0.923489	-2.900930
127	1	0	5.737466	-0.558895	-3.856602
128	1	0	4.223903	0.350063	-3.794115

----

3. References:

[1] A. B. Pangborn, M. A. Giardello, R. H. Grubbs, R. K. Rosen, F. J. Timmers, *Organometallics* **1996**, *15*, 1518–1520.

[2] G. A. Morris, R. Freeman, *J. Am. Chem. Soc.* **1979**, *101*, 760–762.

[3] B. J. Helmer, R. West, *Organometallics* **1982**, *1*, 877–879.

[4] SAINTPLUS: Software Reference Manual, Version 6.45, Bruker-AXS, Madison, WI, 1997-2003.

[5] R. H. Blessing, *Acta Cryst. A* **1995**, *51*, 33–38.

[6] SADABS: Version 2.1 Bruker-AXS: Madison, WI 1998.

[7] G. M. Sheldrick, *Acta Cryst. A* **2008**, *64*, 112–122.

[8] W. J. Leigh, C. R. Harrington, I. Vargas-Baca, *J. Am. Chem. Soc.* **2004**, *126*, 16105–16116.

[9] R. Fischer, D. Frank, W. Gaderbauer, C. Kayser, C. Mechtler, J. Baumgartner, C. Marschner, *Organometallics* **2003**, *22*, 3723–3731.

[10] J. Hlina, J. Baumgartner, C. Marschner, P. Zark, T. Müller, *Organometallics* **2013**, *32*, 3300–3308.

[11] R. Fischer, T. Konopa, S. Ully, J. Baumgartner, C. Marschner, *J. Organomet. Chem.* **2003**, *685*, 79–92.

[12] N. Kuhn, T. Kratz, *Synthesis* **1993**, 561–562.

[13] J. Zeitouny, V. Jouikov, *Phys. Chem. Chem. Phys.* **2009**, *11*, 7161–7170.

[14] C. K. Mann, K. K. Barnes, *Electrochemical reactions in nonaqueous systems*; M. Dekker, 1970.

[15] M. J. Frisch, G. W. Trucks, J. R. Cheeseman, G. Scalmani, M. Caricato, H. P. Hratchian, X. Li, V. Barone, J. Bloino, G. Zheng, T. Vreven, J. A. Montgomery, G. A. Petersson, G. E. Scuseria, H. B. Schlegel, H. Nakatsuji, A. F. Izmaylov, R. L. Martin, J. L. Sonnenberg, J. E. Peralta, J. J. Heyd, E. Brothers, F. Ogliaro, M. Bearpark, M. A. Robb, B. Mennucci, K. N. Kudin, V. N. Staroverov, R. Kobayashi, J. Normand, A. Rendell, R. Gomperts, V. G. Zakrzewski, M. Hada, M. Ehara, K. Toyota, R. Fukuda, J. Hasegawa, M. Ishida, T. Nakajima, Y. Honda, O. Kitao, H. Nakai, *Gaussian 09*.

[16] Y. Zhao, D. G. Truhlar, *Theor. Chem. Acc.* **2007**, *120*, 215–241.

[17] K. Fukui, *Acc. Chem. Res.* **1981**, *14*, 363–368.

[18] H. P. Hratchian, H. B. Schlegel, In *Theory and Applications of Computational Chemistry*; Dykstra, C. E.; Frenking, G.; Kim, K. S.; Scuseria, G. E., Eds.; Elsevier: Amsterdam, 2005; pp. 195–249.

[19] R. Bauernschmitt, R. Ahlrichs, *Chem. Phys. Lett.* **1996**, *256*, 454–464.

[20] R. E. Stratmann, G. E. Scuseria, M. J. Frisch, *J. Chem. Phys.* **1998**, *109*, 8218.

[21] F. Weigend, R. Ahlrichs, *Phys. Chem. Chem. Phys.* **2005**, *7*, 3297–3305.
